# Sexual Dimorphism in Innate Immunity: The Role of Sex Hormones and Epigenetics

**DOI:** 10.3389/fimmu.2020.604000

**Published:** 2021-01-21

**Authors:** Rebecca Shepherd, Ada S. Cheung, Ken Pang, Richard Saffery, Boris Novakovic

**Affiliations:** ^1^ Epigenetics Group, Infection and Immunity Theme, Murdoch Children’s Research Institute, Royal Children’s Hospital, Parkville, VIC, Australia; ^2^ Department of Paediatrics, The University of Melbourne, Parkville, VIC, Australia; ^3^ Department of Medicine (Austin Health), The University of Melbourne, Parkville, VIC, Australia; ^4^ Department of Endocrinology, Austin Health, Heidelberg, VIC, Australia; ^5^ Brain and Mitochondrial Research, Murdoch Children’s Research Institute, Royal Children’s Hospital, Parkville, VIC, Australia; ^6^ Inflammation Division, The Walter and Eliza Hall Institute of Medical Research, Parkville, VIC, Australia; ^7^ Department of Adolescent Medicine, Royal Children’s Hospital, Parkville, VIC, Australia

**Keywords:** epigenetics, innate immunity, progesterone and estradiol, pregnancy hormones, cross-sex hormone treatment, sexual dimorphism

## Abstract

Sexual dimorphism refers to differences between biological sexes that extend beyond sexual characteristics. In humans, sexual dimorphism in the immune response has been well demonstrated, with females exhibiting lower infection rates than males for a variety of bacterial, viral, and parasitic pathogens. There is also a substantially increased incidence of autoimmune disease in females compared to males. Together, these trends indicate that females have a heightened immune reactogenicity to both self and non-self-molecular patterns. However, the molecular mechanisms driving the sexually dimorphic immune response are not fully understood. The female sex hormones estrogen and progesterone, as well as the male androgens, such as testosterone, elicit direct effects on the function and inflammatory capacity of immune cells. Several studies have identified a sex-specific transcriptome and methylome, independent of the well-described phenomenon of X-chromosome inactivation, suggesting that sexual dimorphism also occurs at the epigenetic level. Moreover, distinct alterations to the transcriptome and epigenetic landscape occur in synchrony with periods of hormonal change, such as puberty, pregnancy, menopause, and exogenous hormone therapy. These changes are also mirrored by changes in immune cell function. This review will outline the evidence for sex hormones and pregnancy-associated hormones as drivers of epigenetic change, and how this may contribute to the sexual dimorphism. Determining the effects of sex hormones on innate immune function is important for understanding sexually dimorphic autoimmune diseases, sex-specific responses to pathogens and vaccines, and how innate immunity is altered during periods of hormonal change (endogenous or exogenous).

## Introduction

The innate immune system is comprised of several physical, chemical, and cellular mechanisms which serve as the first line of defense against pathogens (1). Cells of the innate immune system include monocytes, macrophages, neutrophils, dendritic cells (DCs), natural killer (NK) cells, eosinophils, and basophils (1). The innate immune response is influenced by a range of intrinsic (host) and extrinsic (environmental) factors, which can affect susceptibility to infection. Twin studies suggest a heritability of circulating inflammatory markers, such as c-reactive protein, between 20%–56% (2, 3) and thus attribute a large proportion of the variation to environmental exposures.

It is well established that a key protective aspect of the adaptive immune system is the genetically driven capacity to “remember” specific exposures and mount heightened responses to subsequent exposures. Interestingly, innate immune cells also have the capacity to develop a “memory” in response to specific exogenous exposures *via* specific metabolic and epigenetic reprogramming (4), with wide-ranging implications for complex disease and infection response (5). This phenomenon, termed Trained Immunity (TRIM), was originally identified in human monocytes in response to microbe exposures (6, 7), but has subsequently been associated with certain metabolites and danger signals (8–10).

Sex is an important influence on the innate immune system. This influence arises not only due to genetic differences between males and females but also due to differences in sex hormones that alter the environmental milieu to which immune cells are exposed. Consistent with this, sexual dimorphism exists in a range of immune processes, including an individual’s response to pathogens and vaccines. For example, a systems immunology approach of 534 healthy individuals showed that sex and age, along with season influence the *ex vivo* inflammatory response of monocytes to multiple microbial stimuli (11). Females also demonstrate reduced infection rates for a variety of bacterial, viral, and parasitic infections, including *Helicobacter pylori* (12), *Mycobacterium tuberculosis* (13), hepatitis B virus (14), and *Aspergillus fumigatus* (15), while a recent analysis of COVID-19-related deaths among 17 million adults demonstrated that being female was a strong protective factor (hazard ratio of being male: 1.59) (16). Similarly, females display a stronger immune response to some vaccines, including the trivalent influenza virus and hepatitis B virus vaccines [reviewed by Klein et al. (17)]. As another example, pregnancy induces a broad range of maternal innate immune adaptations (18), some of which are remembered beyond parturition (19) and explained at least in part by changes in the hormonal milieu.

In this review, we will discuss the ability of sex hormones to alter mammalian innate immune phenotypes through epigenetic remodeling. This review will discuss the effects of sex hormones (estrogen, progesterone and androgens) on innate immune function, the potential role of sex hormones in autoimmunity, and the transcriptomic or epigenetic changes observed across hormonal shifts (including puberty, pregnancy, menopause, menopausal hormone therapy, and gender-affirming hormone therapy).

## Sex Hormone Associated Molecular Reprogramming of Innate Immunity

Female sex hormones, such as estrogen and progesterone, and male sex hormones, such as testosterone and other androgens, are steroid hormones that modulate a wide range of biological processes, including various aspects of innate immune system functioning (20).

**Figure 1:**
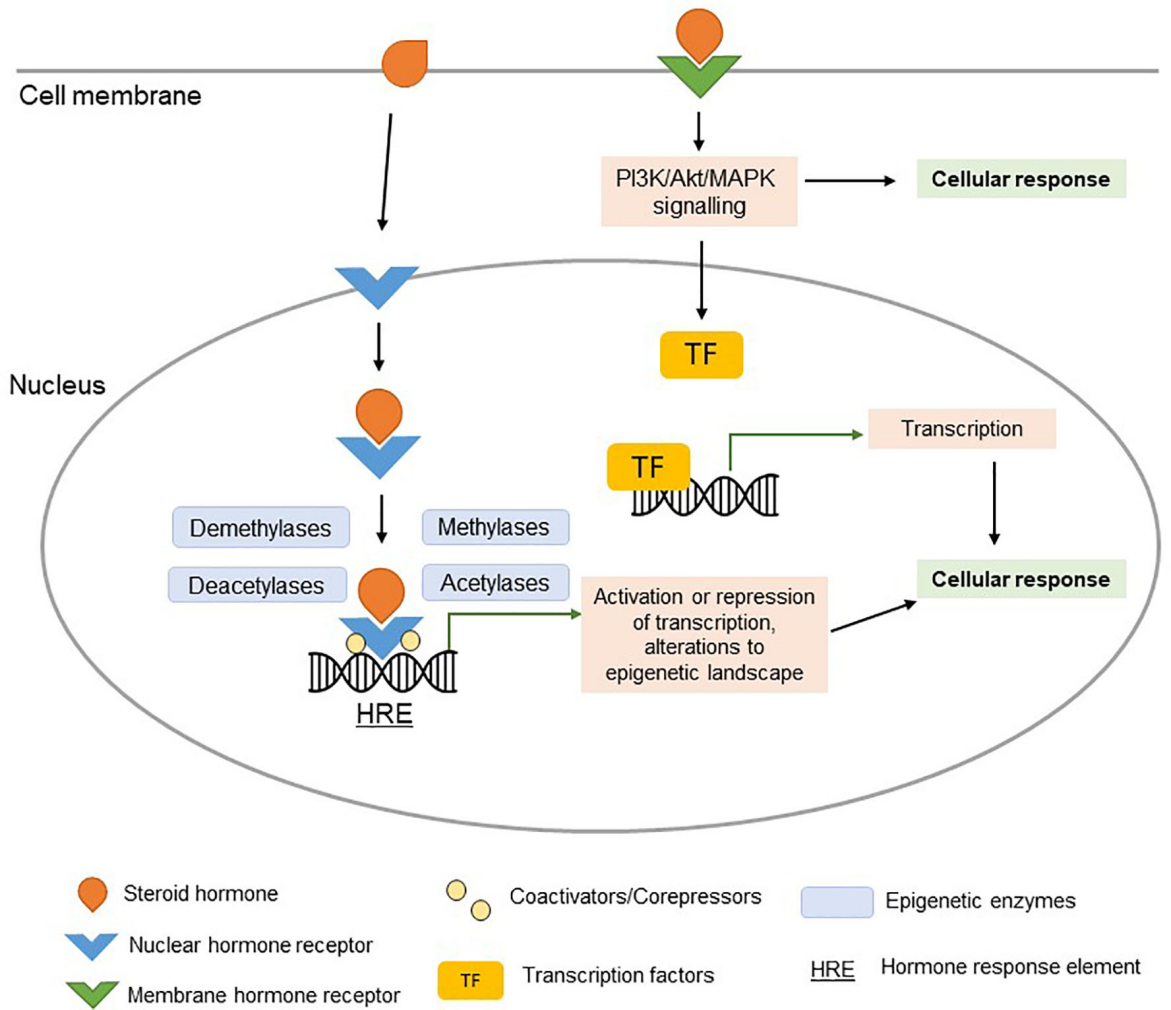
Steroid hormone signaling. Ligand-bound nuclear hormone receptors bind to hormone response elements (HREs) in the promoters of target genes (genomic signaling). Coactivators, corepressors, and epigenetic regulatory enzymes interact with ligand-bound nuclear hormone receptors, regulating their effect on transcription. The activation or repression of target genes (potentially orchestrated by epigenetic changes) can alter the cellular response in a hormone-dependent manner. Signaling of steroid hormones can occur rapidly *via* membrane hormone receptor signaling (non-genomic signaling), resulting in activation of PI3K/Akt/MAPK pathways and downstream TF signaling pathways.

Estrogen, progesterone, and testosterone interact with nuclear hormone receptors (estrogen receptor (ER), progesterone receptor (PR), and androgen receptor (AR), respectively) in a wide variety of cell types, including immune cells. Ligand-bound nuclear hormone receptors have a high affinity for specific sequences of DNA known as hormone response elements (HREs) located in promoters of target genes (**Figure 1**) (21). Thus, numerous genes are, at least in part, regulated by sex hormones. Additionally, sex hormones can also influence gene expression through other mechanisms, including G-protein coupled receptor signaling, and rapid membrane signaling (22). Although beyond the scope of this review, it is important to note that sex hormone receptors may also function through ligand-independent signaling.

The ER has two major isoforms: the alpha receptor (encoded by *ESR1* on chromosome 6) and the beta receptor (encoded by *ESR2* on chromosome 14). ER-α and ER-β are widely expressed in human immune cells, including cells of the innate immune system (23–26). ERs, when bound to their estrogen ligand, function as transcription factors by directly binding to estrogen response elements (EREs) in gene promoters, thus inducing or inhibiting transcription (27, 28). Additionally, ligand-bound ERs interact with other transcription factors, co-regulators, and co-repressors, and therefore can also indirectly influence downstream transcription (27, 28). This is directly relevant to various inflammatory pathways, with a number genes encoding for cytokines, chemokines, and cell surface immune markers having been shown to be regulated by estrogen signaling [reviewed by Khan & Ahmed (29)]. For example estrogen has been shown to have a strong influence on NF-κB signaling, which plays a key role in a variety of inflammatory and autoimmune processes (30–36).

Progesterone is capable of binding to progesterone receptors (PRs) and glucocorticoid receptors (GRs). Much like the nuclear ER, the nuclear PR has two isoforms nPR-α and nPR-β. However, these isoforms are encoded by a single gene (*PGR)* on chromosome 11 (37). In addition to nuclear PRs, progesterone can also signal through membrane PRs (38). A variety of human and murine immune cells have been shown to express PRs, including cells of the innate immune system (39, 40). The expression of PRs in immune cells may vary between sexes, with one study demonstrating that murine female-derived DCs express higher amounts of PRs compared to male-derived DCs (41). The GRs are more abundant in immune cells compared to PRs, and thus progesterone-induced GR signaling may act as an alternate pathway by which progesterone can modify immune function (42, 43). Indeed, in murine macrophages and DCs, progesterone attenuated LPS and poly I:C-induced IL-6 production exclusively through GR signaling, whereas LPS-induced IL-12p40 production was attenuated by progesterone *via* GR signaling or PR signaling (or both) (43, 44).

The actions of testosterone largely occur through androgen receptor (AR) signaling, with its derivative DHT being a more potent agonist. The AR is encoded by the *AR* gene on the X chromosome. When not bound to an androgen ligand, the AR resides in the cytoplasm bound by heat shock proteins (HSPs) and chaperone proteins (45). Upon interaction with an androgen ligand (such as testosterone or DHT), the AR is released from the HSPs and chaperone proteins and the ligand-bound AR translocates to the nucleus (45). In the nucleus, the ligand-bound AR binds to androgen response elements (AREs) and modulates gene expression of target genes, facilitated by coactivators and corepressors (45). The AR has been shown to be expressed in a number of immune cells in human and murine models [reviewed by Bupp and Jorgensen (46)], including cells of the innate immune system.

### Androgens

A number of human and murine studies have shown an overall anti-inflammatory effect of testosterone, which may contribute to the dampened immune response to infection and vaccination in males. For example, 5-lipoxygenase (5-LO) is a key enzyme involved in the synthesis of leukotrienes, which are pro-inflammatory mediators with potent vasoconstricting and chemoattractant properties (47). Pergola et al. demonstrated that female-derived human monocytes have a higher (1.8-fold) 5-LO product formation than that of male-derived monocytes, and then went on to show that *in vitro* stimulation of female-derived monocytes with the testosterone metabolite 5-α-dihydrotestosterone (DHT) (10 nM) suppressed the synthesis of 5-lipoxygenase (5-LO) products (47). Testosterone also appears to dampen the cytokine response, with 10 nM of testosterone attenuating IFN-γ-induced (500 U/ml) *TNFA* mRNA expression in THP-1 monocytes (48) and 1 x 10^-6^ M of testosterone attenuating LPS-induced TNF production in murine macrophage-like cells (RAW 264.7) (49). Conversely, one study found that *in vitro* stimulation of female-derived human monocytes with physiological levels of testosterone (2 x 10^-7^ to 2 x 10^-9^ mol/L) resulted in an elevation of IL-12 and IL-1β-producing monocytes following lipopolysaccharide (LPS) stimulation, but had no effect on TNF-α-producing monocytes (50). In human male-derived monocytes, *in vitro* exposure to dihydrotestosterone (DHT), reduced BCG-induced TNF-α production at a concentration of 100 pmol/ml, and reduced BCG-induced IL-6 production at concentrations of 10 and 100 pmol/ml (51). This effect was not observed in female derived human monocytes (51). Toll-like receptor (TLR) 4, a pattern recognition receptor for LPS, appears to be under regulation by testosterone, with *in vitro* testosterone stimulation (1 x 10^-6^ M for 24 h) reducing cell surface TLR4 expression in a murine macrophage cell-line (RAW 264.7) and in macrophages isolated from orchiectomized mice (49). Congruently, an *in vivo* murine approach demonstrated that orchiectomized male mice exhibit significantly increased cell surface TLR4 expression (compared to sham orchiectomized male mice and non-orchiectomized male mice), and show increased susceptibility to LPS-induced shock (49). Importantly, when orchiectomized male mice were given exogenous testosterone treatment, the significant increase in TLR4 surface expression was not observed, and the increased susceptibility to endotoxin shock was abolished (49). Together, these *in vitro* human immune cell studies and murine studies indicate an immunomodulatory effect of androgens.

### Estrogens

By contrast, studies investigating the effects of estrogen on immune cells show varied results, likely due to the fact these hormones have been studied more extensively. The overall effect appears to depend on the form of estrogen, its concentration (physiological versus supraphysiological and pregnancy-associated levels), cell type, sex, and the relevant receptor signaling pathways. Moreover, further complexity arises in association with the varying female hormonal milieu across the estrous cycle, with serum estrogen (estradiol) peaking during the ovulatory phase and serum progesterone peaking during the luteal phase (52).

In general, low level (physiological) estradiol enhances the pro-inflammatory capacity of human and murine macrophages and monocytes, whereas supraphysiological (late-pregnancy-associated) estradiol levels suppress their pro-inflammatory capacity [reviewed by (53)]. This is also reflected in the sustained anti-inflammatory shift observed in maternal macrophages and monocytes in mid to late pregnancy (discussed in a later section) (42). Indeed, murine macrophages exposed *in vitro* to low level estradiol (4.17 x 10^-11^ M, 4.17 x 10^-10^ M and 2.09 x 10^-9^ M) for 16 h attenuated LPS-induced TNF-α production, and 4.17 x 10^-10^ M estradiol suppressed LPS-induced *TNFA, IL1* and *IL6* gene expression (54). Consistent with this, *in vitro* exposure of male-derived human monocytes to low levels of estradiol (10^-9^ to 10^-10^ M) resulted in maximal IL-1 activity, whereas higher levels of estradiol (10^-7^ M) reduced IL-1 activity (55). Similarly, *in vitro* exposure of the monocytic cell line THP-1 [differentiated by 12-O-tetradecanoylphorbol-13-acetate (TPA) for 48 h] to low level estradiol (10^-9^ M) in the final 20 h of differentiation resulted in increased expression of LPS-induced *IL1A* and *IL1B* mRNA (56). In human peripheral blood mononuclear cells (PBMCs), *in vitro* estradiol stimulation had a sex-specific effect. Intriguingly, estradiol (1.25 x 10^-10^ M to 1.25 x 10^-7^ M) triggered TNF-α and IL-6 production in male-derived PBMCs but not female-derived PBMCs (57). Co-stimulation of PBMCs with estradiol and LPS attenuated the LPS-induced TNF-α response in PBMCs derived from both sexes at concentrations of 1.25 x 10^-8^ M and 1.25 x 10^-7^ M estradiol (and also with 1.25 x 10^-10^ M and 1.25 x 10^-9^ M estradiol in male-derived PBMCs) (57). The presence of estradiol also influences the *in vitro* Bacillus Calmette-Guerin (BCG)-induced cytokine response in human monocytes. In particular, estradiol reduces the TNF-α response in monocytes derived from both males and females, as well as the IL-6 and IL-10 response in male monocytes, following a 6-h BCG stimulation in the presence of estradiol (1 x 10^-10^ M to 1 x 10^-8^ M) compared to BCG alone (51).

Estrogen has been shown to be implicated in neutrophil apoptosis, chemotaxis, and the formation of neutrophil extracellular traps (NETs), which are extracellular chromatin fibers capable of binding pathogens, resulting in cell death (NETosis) (58). Reduced spontaneous apoptosis in female-derived neutrophils compared to male-derived neutrophils has been observed (59). Moreover, in neutrophils derived from both sexes, treatment with physiological estrogen (and physiological progesterone) resulted in a further delay in spontaneous apoptosis in a cytochrome c-mediated manner (59). Murine *in vivo* estrogen treatment attenuated mRNA expression of inflammatory mediators (adhesion molecules, chemokines, and cytokines) in a model of acute artery injury, resulting in reduced neutrophil chemotaxis (60), suggesting a vasoprotective role of estrogen. In a human neutrophil-like cell line (HL-60), estradiol increased the formation of neutrophil extracellular traps (NETs) *via* the ER and G-protein coupled receptor.

In a murine model, the *in vivo* removal of endogenous estrogen has been shown to reduce the inflammatory response. Rettew et al. demonstrated that ovariectomized mice have reduced serum TNF-α, IL-6, and IL-10 levels following a sublethal *in vivo* LPS challenge, compared to sham ovariectomized mice (no differences in severity of endotoxin shock) (61). Moreover, the ovariectomized mice showed reduced serum lipopolysaccharide binding protein (LBP) levels, and reduced cell surface TLR4 and LPS-binding activity in isolated peritoneal monocytes/macrophages. Intriguingly, the exogenous replacement of estradiol (at supraphysiological levels) in ovariectomized mice drastically increased the severity of endotoxin shock, elevated serum LBP and TNF-α, and increased surface TLR4 and LPS-binding in isolated peritoneal monocytes/macrophages compared to both ovariectomized mice and sham ovariectomized mice (61). These findings further suggest a dual role of estradiol at physiological versus supraphysiological levels. Understanding the influence of estradiol on the response to bacterial stimuli is significant in the context of sepsis, as it may explain why females generally have a better sepsis outcome compared to males (62, 63).

### Progesterone

Several studies have demonstrated an overall suppressive effect of progesterone on innate immune cells (64). In contrast to estrogen, progesterone has been shown to have a suppressive effect on NET formation and NETosis, and can diminish the pro-NETotic effect of estrogen (65). An *in vitro* study of murine macrophages demonstrated that progesterone suppressed the inflammatory response, with lower arginase and inducible nitric oxide synthase 2 activity, in a dose-dependent manner, in response to exposure to LPS (200 ng/ml), IL-4 (100 U/ml), or a combination of both (66). In agreement, Jones et al. demonstrated that LPS-induced nitric oxide production is reduced by progesterone (7.8 × 10^-6^ M to 6.25 × 10^-5^ M) in male murine bone-marrow derived macrophages (44). Additionally, the authors reported a reduction in LPS-induced (12.5 µg/ml, 72 h) IL-12p40 production in murine bone-marrow derived macrophages exposed to 1 x 10^-7^ M to 8 x 10^-6^ M of progesterone. In a follow-up study, Jones et al. demonstrated that progesterone was able to inhibit LPS-induced (800 ng/ml, 72 h) IL-6 production at 3.125 × 10^-5^ M and 6.15 × 10^-5^ M, and poly I:C-induced (50 µg/ml, 72 h) IL-6 production in a dose-dependent manner (5.0 × 10^-7^ M to 6.25 × 10^-5^ M) in male murine bone marrow-derived DCs (43). Similarly, progesterone had a suppressive effect on LPS-induced and poly I:C-induced IL-12p40 production in murine DCs (43). In both male- and female-derived human primary monocytes, 1.0 × 10^-6^ M progesterone suppressed the formation of 5-LO products in response to LPS/N-formyl peptide (67).

Interestingly, like estradiol, the effects of progesterone on innate immune cells may diverge depending upon its concentration. Thus, at very low doses (10^-9^ M), progesterone (48 h *in vitro* incubation) enhanced IL-1 activity, whereas at much higher doses (10^-7^ to 10^-5^ M) progesterone reduced IL-1 activity in human male-derived peripheral monocytes (55), consistent with the studies described above. In this way, the suppressive effects of progesterone on innate immunity—which are likely to influence the response to infection—may be more likely to be observed during particularly physiological states, including the luteal phase of the menstrual cycle (when progesterone is the dominant hormone) or pregnancy (when progesterone (and estradiol) levels greatly exceed those of non-pregnant individuals).

## The Role of Sex Hormones in Autoimmunity

Autoimmunity refers to a loss of self-tolerance and state of immune reactivity towards self-antigens. Autoimmunity can result in damage to tissue, and a disease state that is caused by autoimmunity is termed an “autoimmune disease”. Although self-reactive T and B lymphocytes have traditionally been the focus in autoimmune disease, there is now a large body of evidence demonstrating a role for innate immune cells in autoimmune disease (68). For instance, macrophages have a multifunctional role in autoimmunity, not only producing potent inflammatory cytokines and mediators which influence the local tissue microenvironment, but also presenting antigens to lymphocytes (thus bridging the innate and adaptive immune systems) (69).

There is considerable sexual dimorphism in the incidence of many autoimmune diseases. Females display increased susceptibility to systemic lupus erythematosus (SLE), Sj̈ogren’s syndrome, scleroderma, myasthenia gravis, Grave’s disease, rheumatoid arthritis and multiple sclerosis [reviewed by Rubtsova et al. (70)]. Indeed, it is estimated that females account for more than 78% of all cases of autoimmune diseases (71). However, the mechanisms that drive sexual dimorphism in autoimmunity are yet to be fully elucidated. Potential mechanisms include the function and downstream effects of genes encoded by the X or Y chromosomes, the effects of sex hormones on immune cell function, and environmental factors such as differential responses to infection and gut microbial composition (70). Although autoimmune diseases can occur at any stage of life, many autoimmune diseases with a female preponderance more commonly arise in the reproductive rather than pre-pubertal years (72, 73). Moreover, the severity of many autoimmune diseases changes in synchrony with periods of major endocrine change, such as pregnancy and menopause (72, 74). In this way, indirect evidence suggests that female sex hormones are likely to be a key driver for the sex bias seen in autoimmunity.

Consistent with this, numerous studies in both animals and humans have now directly implicated sex hormones in autoimmunity. Estrogen signaling can have a protective or detrimental role in autoimmunity. In a murine model of lupus, ER-α deficiency resulted in improved disease measures (75). Signaling through ER-β in a murine model of autoimmune thyroiditis was shown to have a disease-aggravating effect. However, ER-α signaling has a beneficial anti-inflammatory effect in a murine models of arthritis (76) and multiple sclerosis (77). In this way, murine studies have highlighted an important role for estrogen signaling in autoimmunity, but further studies are required to understand the specific mechanistic pathways.

Testosterone appears to have a protective effect against autoimmunity, with a substantial body of evidence now having demonstrated an overall anti-inflammatory effect [reviewed by Bianchi et al. (78)]. In murine studies, the protective role of testosterone in autoimmunity has also been demonstrated in models of lupus (79), type I diabetes (80), and arthritis (81). In human cells, *in vitro* testosterone stimulation resulted in a significant decrease in IgG and IgM production by PBMCs, and a reduced IL-6 production by monocytes (82). This effect has also been observed in SLE-derived immune cells, with testosterone suppressing total IgG and anti-dsDNA IgG in SLE-derived PBMCs, and suppressing IL-6 production in SLE-derived monocytes (83). Testosterone deficiency or a reduced androgen to estrogen ratio has been demonstrated in numerous (but not all) studies in men diagnosed with female-biased autoimmune diseases, including rheumatoid arthritis (84–91), systemic lupus erythematosus (92–101), and multiple sclerosis (102, 103), as reviewed by Bove (74). However, since testosterone concentrations vary significantly with the presence of acute or chronic disease, these lowered testosterone concentrations might simply be a reflection of illness rather than the driver of immune dysfunction. Nevertheless, a large-scale longitudinal study demonstrated that untreated hypogonadism in men increases the risk of both lupus and rheumatoid arthritis (104). Moreover, men with autoimmune thyroid disease and diffuse cutaneous systemic sclerosis have been shown to have higher levels of circulating estradiol compared to unaffected males (105–107). Together, these studies indicate a protective role of testosterone in autoimmunity.

## A Sex-Specific Immune Transcriptome

While studies looking at sex-specific differences in the transcriptome of innate immune cells specifically are lacking, several studies have compared female and male transcriptomes in whole blood (108–111). Jansen et al. (109) measured sex-specific differences in gene expression *via* microarray in 5,241 individuals. The authors identified 582 genes that were influenced by sex in peripheral blood (109), and these genes were associated with responses to cytokines, type I interferon signaling and rheumatoid arthritis (109). The sex-specific differences in gene expression were more pronounced in women using oral contraceptives and less pronounced in post-menopausal women, thus supporting a role for estrogen in the establishment or maintenance of the sexually dimorphic blood transcriptome (109). In an integrative multi-cohort approach (3,672 individuals across 28 studies), Bongen et al. identified 144 differentially expressed genes between females and males (aged 18 to 40) in healthy adult blood (108). Importantly, three quarters of the identified genes were autosomal, indicating that a sex-specific blood transcriptome extends far beyond the X and Y chromosomes (108). A number of female-enriched genes highly expressed in CD4 T-cells indicated an enhanced adaptive immune response. By contrast, a number of male-enriched genes highly expressed in myeloid cells (monocytes/macrophage and neutrophil/basophil clusters), indicated enhanced aspects of innate immunity, such as phagocytosis and anti-microbial defenses (108). The pattern of both differentially expressed autosomal and allosomal genes (X and Y) reliably and independently distinguished between sexes in validation cohorts. In mice, a sex-specific transcriptome has been identified in peritoneal cavity-derived macrophages, splenic macrophages, and microglia (110). Female-enriched pathways included the response to interferon, complement, IL-6/JAK/STAT pathways and coagulation pathways (110). Female-enriched genes that are associated with immune pathways, adaptive immunity, and estrogen regulation warrant further investigation, as they may provide insight into the pathways driving the sexually dimorphic immune and autoimmune responses. Moreover, genes related to the response to stimuli may elucidate the mechanisms behind the heightened infection and vaccination response in females.

## Sex Hormones as Epigenetic Modifiers in Innate Immune Cells

The underlying mechanisms that drive innate immune gene expression and phenotypic changes occur at the level of epigenetic marks (112). The term epigenetics means ‘above DNA’ and refers to the study of molecular interactions that influence chromosome structure and gene activity (113). In monocytes/macrophages, lineage-determining transcription factors, such as PU.1 and C/EBPs, establish the regulatory landscape within which stimulus-specific transcription factors can act (114). These regulatory regions are marked by specific ‘active’ posttranslational histone tail modifications, which influence the transcriptional output of the innate immune cells in response to microbial ligands (115–117). DNA methylation, a covalent modification of cytosines in the context of CpG dinucleotides, is also remodeled in monocytes in response to stimulation (116, 118).

Ligand-bound nuclear sex hormone receptors interact with a number of co-regulators (co-activators and co-repressors) which, as a complex, can alter chromatin structure and histone tail modifications, thus facilitating transcriptional activation or repression of target genes (**Figure 1**) (28, 45). Hormone-associated changes in gene expression in innate immune cells are mediated through widespread epigenetic remodeling downstream of hormone receptor signaling pathways, as demonstrated *via in vitro* glucocorticoid receptor signaling studies in human monocytes and macrophages (119).

In cancer cells, estrogen-induced ER signaling has been shown to trigger the re-organization of chromatin through histone tail modifications including methylation, acetylation, and phosphorylation [reviewed by Mann et al. (120)]. Sex-specific open chromatin regions have been identified in murine macrophages (110), suggesting a sexually dimorphic immune epigenome. Indeed, Mamrut et al. investigated the methylome and transcriptome of human B cells, CD4+ T cells, CD8+ T cell, and monocytes in adult males and females (111). Autosomal sex-specific differentially methylated regions were identified and this epigenetic signature was robustly expressed across immune cell types (111). Together, these studies provide nascent evidence for a sex-specific immune transcriptome and methylome, with a clear extension beyond X- and Y genes potentially mediated by the hormonal milieu. *In vitro* stimulation of human endometrial stromal fibroblasts with estrogen and progesterone induced changes to the methylome, transcriptome, and chromatin landscape (121), indicating a direct epigenome-altering effect of female sex hormones. In the context of sexual dimorphism in infection and autoimmunity, the epigenetic effects of estrogen, progesterone, and testosterone in immune cells warrant investigation.

## Immunological, Transcriptome, and Epigenome Differences During Periods of Hormonal Change

**Figure 2:**
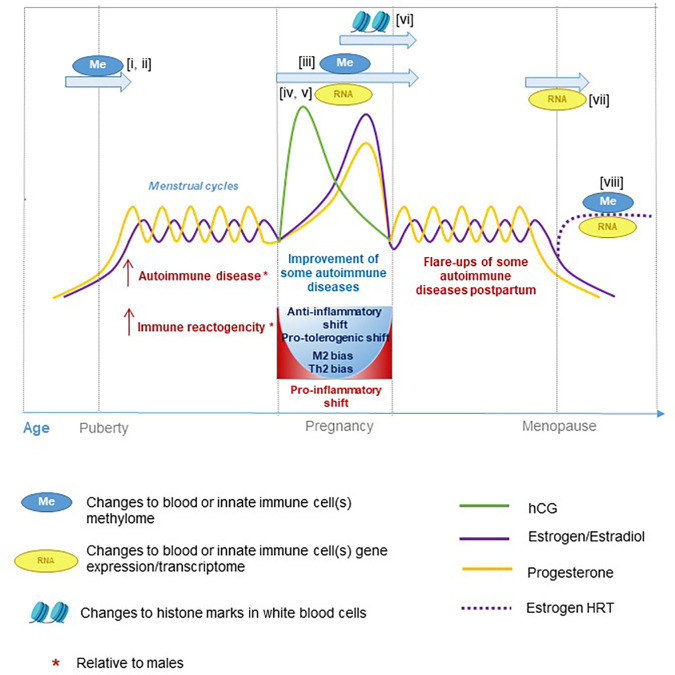
Hormone fluctuations in human females during aging and pregnancy. These hormone fluctuations are associated with immune function and susceptibility to certain inflammatory diseases. Several studies have performed epigenetic and transcriptional profiling at different stages of life: i) (122), ii) (123), iii) (124), iv) (125), v) (126), vi) (127), vii) (128), and viii) (129).

There are distinct temporal changes in hormonal levels throughout the male and female lifespan, some longer term and some cyclical. In females, these include puberty, menopause, pregnancy, and the menstrual cycle. In males, these include puberty as well as a decline in circulating androgen levels with age that occurs at a more gradual rate than menopausal-associated hormonal shifts in females. These periods of hormonal change also coincide with significant transcriptional, epigenetic, and immunological variation in a range of cell types and tissues (**Figure 2**).

### Puberty

At the onset of puberty, profound and distinct hormonal changes occur in males and females. Puberty marks the initiation of increased testosterone production in males, and increased estrogen and progesterone production in females. Immunological changes also occur during puberty, with adolescents showing an increased percentage of NK cells compared to pre-pubertal children in both sexes (130), with another increase observed in the elderly. Moreover, sex-specific differences among adolescents have been observed, with adolescent females having a lower percentage of monocytes (130) and CD19+ B-cells (131) and a higher percentage of T-cells (CD3+) and CD4+ T-cells (132) compared to males. In almost all of these examples, puberty as a group shows an intermediate profile between infancy and older age, suggesting these changes are not transient, but progressive.

There is an absence of longitudinal studies investigating a sex-specific transcriptome across puberty and adolescence, but DNA methylation studies (122, 123) indicate the formation of a sex-specific methylome during puberty, with sex hormone signaling (particularly estrogen signaling) playing a central role. Thompson et al. reported sex-specific DNA methylation changes in human PBMCs between pre-puberty and post-puberty (8 and 14 years old, respectively) (122). The female differentially methylated probes (DMPs) between pre-puberty and post-puberty were over-represented for estrogen response elements (ERE) (122), suggesting an interplay between epigenetic, transcriptional, and hormonal regulation. Genes located near the 347 female DMPs were enriched for hormone receptor signaling and immune pathways, whereas genes near the 50 male DMPs were enriched for adrenaline and noradrenaline synthesis (122). In a similar longitudinal study, Almstrup et al. investigated DNA methylation changes in peripheral blood across puberty, with males and females analyzed together (123). Of the identified DMPs, a subset (94 DMPs) separated nearly all samples into pre-pubertal and post-pubertal states, and a second subset (133 DMPs) was associated with three or more hormones in males, suggesting that the methylation signature in blood reflects both hormone levels and pubertal age. Testosterone level were specifically associated with 999 DMPs, which was higher than for other circulating hormones tested. There was substantial overlap between the puberty-predictive (29 percent) and hormone-predictive CpGs (27 percent) identified by Almstrup et al. (123) and the female DMPs identified by Thompson et al. (122).

Testosterone Through Life and Epigenetics/RNA

https://www.ncbi.nlm.nih.gov/pmc/articles/PMC5075254/

https://www.nature.com/articles/s41598-018-25694-0.pdf?origin=ppub (immunocompetence hypothesis)

https://onlinelibrary.wiley.com/doi/abs/10.1002/ajhb.20419

https://www.ncbi.nlm.nih.gov/pmc/articles/PMC5109279/

https://files.sld.cu/inmunologia/files/2014/01/2014-01-13-testosterone.pdf

https://www.jimmunol.org/content/198/5/1782

It remains unclear whether puberty-associated hormonal changes drive DNA methylation changes, or alternatively, whether puberty-associated methylation changes influence hormone levels. An integrative, longitudinal approach to analyzing the hormonal milieu alongside the immune transcriptome, epigenome and proteome across puberty will provide further understanding of sex-specific immunological differences.

### Pregnancy

Profound hormonal, immunological, transcriptomic, and epigenetic changes occur during pregnancy. Human chorionic gonadotropin (hCG), typically undetectable in non-pregnant individuals, is initially produced by the implanted conceptus and later by the placenta (133). Serum hCG peaks in the first trimester of pregnancy and hCG plays a pivotal role in implantation, placentation, and the establishment of feto-maternal tolerance (133–135). Progesterone and estradiol, normally produced by the corpus luteum of the ovary in non-pregnant females, are produced in high levels by the placenta in the second and third trimesters of pregnancy (133). Estriol, a type of estrogen produced only in pregnancy, is synthesized by the fetoplacental unit (42).

Immunological changes occur throughout pregnancy, both in the periphery and at the feto-maternal interface, orchestrated by the maternal hormonal milieu. Tolerance towards the semi-allogeneic fetus is required to prevent spontaneous rejection, and thus it is advantageous for the maternal immune system to shift towards an anti-inflammatory state (42). This anti-inflammatory shift is observed in T-helper cells, with an overall Th2 bias observed in pregnancy (136–138). The Th2 skew may be regulated by progesterone-receptor signaling, as progesterone triggers the production of progesterone-induced blocking factor (PIBF), which promotes the development of Th2 cells (139). A shift towards a type 2 cytokine environment and a reduction in type 1 cytokines has been shown to be important for a successful pregnancy (140–143). Additionally, pregnancy results in an increased proportion of regulatory T cells (T-regs), promoting implantation and tolerance towards the fetus (144–146). Studies have demonstrated that hCG promotes the development of T-regs (147, 148), and that T-regs are attracted to hCG-producing trophoblasts (149). Moreover, recent evidence shows that hCG can act as an epigenetic modifier (150). Indeed, hCG inhibits CXCL10 expression in decidual stromal cells through histone methylation (H3K27me3) (150). This downregulation of CXCL10 is thought to be important for establishing feto-maternal tolerance by reducing CD8+ T cell attraction (150).

A progressive tolerogenic shift throughout pregnancy has also been observed in maternal innate immune cells. At the feto-maternal interface, decidual macrophages take on an M2-like phenotype (151, 152). In a longitudinal study of maternal PBMCs, Pflitsch et al. demonstrated that the percentage of classical monocytes (CD14^high^CD16^neg^) decreases as pregnancy progresses, while the percentage of intermediate monocytes (CD14^high^CD16^pos-^) increases (153). Within monocyte subsets, maternal serum hCG level was associated with specific surface markers (CD116, CD11b, CCR2). *Ex vivo* studies indicate that maternal monocytes and macrophages have a reduced capacity to produce pro-inflammatory cytokines in response to LPS as pregnancy progresses (153–155). One *ex vivo* study demonstrated that the reduction in LPS-induced TNF-α-positive maternal monocytes was associated with increasing plasma progesterone and estradiol levels (154). This effect may be driven by the ability of estradiol to downregulate NF-κB signaling in myeloid cells (30–32, 54). Conversely, one study found no significant changes in the percentage of TNF-α, IL-1β, or IL-6-positive monocytes between pregnant and non-pregnant individuals following *in vitro* whole blood LPS stimulation (156). They did, however, observe an increased percentage of IL-12-positive monocytes (in LPS-induced and unstimulated conditions) in pregnant women (156). Pregnancy-derived serum can alter the cytokine profile of non-pregnancy-derived cells, with increased IL-10 production and decreased the IL-1β production in human macrophages incubated with pregnancy-derived serum compared to non-pregnancy-derived serum (157). Overall, evidence thus suggests that the maternal immune system increases anti-inflammatory factors and attenuates pro-inflammatory factors as pregnancy progresses (42). Uterine NK cells play a key role in the vascular remodeling of endometrial tissue in the first trimester of pregnancy, and are thought to be under regulation of estrogen. Indeed, Gibson et al. reported that primary uterine NK cells derived from first trimester decidua demonstrate increased migration when treated *ex vivo* with estrone or estradiol (measured by Transwell migration assay and time-lapse microscopy) (158). This effect was abrogated by the estrogen receptor antagonist ICI, further supporting the role of estrogen in uterine NK cell migration. Moreover, expression of *CXCR4* and *CCL2* was upregulated by estradiol, proposing a mechanism for the migration-enhancing and pro-angiogenic effects of estrogen (158). Despite the absence of progesterone receptors, progesterone may regulate uterine NK cells indirectly. For example, *in vitro* treatment of human endometrial stromal cells triggers the secretion of IL-15 in a dose-dependent manner (10^-8^ to 10^-6^ M), which is a known mediator of uterine NK development and survival (159, 160). Together, these findings suggest that estradiol and progesterone regulate the migration, development, and function of uterine NK cells in pregnancy.

Pregnancy-associated immunological adaptations consequently affect maternal immunity and autoimmunity. Pregnant females are more susceptible to severe infection from several pathogens, including the influenza virus, *Toxoplasma gondii*, and malarial *Plasmodium* parasites [reviewed by Robinson et al. (42)]. Patterns in autoimmune disease severity in pregnancy are congruent with the pregnancy-associated Th2 bias. Th1-type autoimmune diseases including rheumatoid arthritis (161), multiple sclerosis (162), and Grave’s disease (163) have been reported to ameliorate during pregnancy and worsen in the post-partum period. By contrast, systemic lupus erythematosus, which is classically regarded as Th2-type autoimmune disease, can increase in severity during pregnancy (164–166).

In light of the phenotypic and functional changes to maternal immune cells, several studies have also investigated the blood transcriptome (125, 126, 167), epigenome (124, 127) and proteome (168) across pregnancy. Longitudinal transcriptome analysis of whole blood in healthy pregnancy revealed sustained downregulation of interferon response and plasma cell signatures, and sustained upregulation of neutrophil and erythropoiesis signatures, with changes observable at less than 16 weeks’ gestation (126). In agreement, Gomez-Lopez et al. observed progressive downregulation of a number of immunoglobulin genes and a reduced B-cell-specific mRNA signature throughout pregnancy, along with an increased erythroid-cell-specific mRNA signature (125). Additionally, the authors observed a decreased T-cell-specific mRNA signature in early to mid-pregnancy, followed by an increase towards parturition (125). Multiple immune-related and inflammatory pathways were also modulated across pregnancy, suggesting transcriptional reprogramming of the immune system to promote fetal tolerance (125). In an *in vivo* porcine model, the endometrial transcriptomic changes observed on day 12 of pregnancy were similar to those induced by an estradiol infusion (167). Overlapping differentially-expressed genes corresponded to biological processes involved in implantation and embryo-maternal crosstalk, suggesting that estradiol may be an underlying trigger for multiple pregnancy-associated changes (167). Changes to the maternal blood epigenome occur as pregnancy progresses, with a DNA methylation study revealing 196 CpGs showing longitudinal intra-individual changes in methylation across pregnancy, with several genes containing multiple differentially methylated CpGs (124). The vast majority of these CpGs (91 percent) demonstrate decreasing methylation across pregnancy, and these CpGs were overrepresented for biological pathways involving metabolism, insulin signaling, and growth of adipose and mammary gland tissue (124), suggesting that epigenetic remodeling is implicated in pregnancy-associated adaptations. Another study showed that pregnancy (and the early postpartum period) induce dynamic and reversible changes in histone methylation of maternal white blood cells (H3K4, H3K9, H3K27, H3K36 and H3K79) (127).

Together these studies indicate that the stages of pregnancy dynamically remodel the immune transcriptome and methylome, and these changes are mirrored by altered immune cell function. It has been suggested that the internal hormonal milieu regulates these epigenetic changes, but lifestyle changes during pregnancy or feto-maternal microchimerism may also contribute to maternal pregnancy-associated epigenetic changes (124).

### Menopause and Aging

Menopause marks the permanent cessation of the menstrual cycle, and thus the end of the reproductive stage. The menopausal transition (staged as pre-menopause, peri-menopause, menopause, post-menopause) results in profound alterations to the hormonal landscape, with a significant reduction in serum estradiol and progesterone levels and an elevation in serum follicle stimulating hormone (FSH) and luteinizing hormone (LH) (169, 170). Menopause alters the gene expression of peripheral monocytes, with differentially expressed genes linked to ontological categories of cell proliferation, metabolism, immune responses, and transport, among others (128). There is a paucity of longitudinal studies investigating the evolving transcriptome and methylome across menopause, and if conducted, particularly in immune cells, such studies would contribute to improved understanding of how sex hormone deprivation in menopause affects immunity. Menopause has also been associated with epigenetic changes, particularly changes in DNA methylation. Menopausal hormone therapy (MHT) is widely used to prevent post-menopausal osteoporosis (171). Studies have shown that MHT can induce changes in the transcriptome of skeletal muscle cells (172) and the methylome of white blood cells (129). In a monozygotic twin study discordant for the use of MHT, 7855 DMRs were detected in white blood cells (129). These DMRs were linked to 4,044 genes, with five genes (all related to bone density or adiposity) showing differential gene expression (*ACBA1*, *CCL5, FASLG, PPP2R2B*, and *UHRF1*) (129). Congruently, the expression levels of these five genes were associated with clinical measures of bone and adiposity in the participants (129). There is an intimate crosstalk between cells of the immune system and skeletal system, with numerous cytokines and transcription factors shared between the two systems (129). It is thus tempting to speculate that detrimental changes to bone density as a result of menopause (i.e. osteoporosis) may be detected in the blood methylome signature. Indeed, a 2018 study by Cheishvilli et al. identified 77 significant differentially methylated CpG sites in the blood of post-menopausal women with osteoporosis (compared to age-matched healthy post-menopausal women) (173). A subset of the associated genes correlated with bone density measures, and their expression was able to predict osteoporosis (173). Moreover, Reppe et al. identified that a substantial proportion of significantly differentially methylated CpGs in the bone of osteoporotic post-menopausal women (compared to healthy post-menopausal women) were also differentially methylated in the blood (174).

It remains unclear whether the DNA methylation changes observed in the menopausal transition are caused by, or an effect of menopause-associated hormonal changes. The transcriptional and epigenetic changes observed before and after menopause may be driven by changes to the hormone milieu, however, it is important to highlight that they may also be driven by senescence.

Aging in males is associated with a slow decrease in testosterone levels, which is more gradual compared to the faster drop in estrogen in women. Loss of testosterone with age is associated higher levels of inflammatory markers, such as IL6 (175), indicating a potential role in inflammaging—the chronic low-grade inflammation with age (176). Further, testosterone was shown to attenuate influenza vaccine response, with older males responding more strongly than younger males (177). Coupled with the association between DNA methylation and testosterone levels in puberty, it would be interesting to explore the potential role for epigenetic mechanisms in mediating the effects of decreased testosterone in aging and immune function.

### Hormone Therapy in Transgender Individuals

Gender-affirming hormone therapy (GAHT), sometimes referred to as cross-sex hormone therapy, is commonly used among transgender individuals undergoing a medical transition, and also results in profound changes within the internal hormonal environment (178). With GAHT, people assigned male at birth who seek feminization receive exogenous estradiol, often alongside drugs with anti-androgenic effects. By contrast, people assigned female at birth who seek masculinization receive exogenous testosterone. While the initiation of GAHT is a period of profound hormonal and physical change (179), the immunological and epigenetic effects of GAHT have not been well defined. In the context of the sexual dimorphism observed in cisgender (i.e. non-transgender) males and females, it would be useful to understand whether transgender individuals undergoing GAHT immunologically resemble their sex assigned at birth or their transitioned gender. Transgender individuals undergoing GAHT are a unique population, as the proposed chromosomal influences (X and Y) of sexual dimorphism remain unchanged, whereas the proposed hormonal influences of sexual dimorphism are introduced at the commencement of GAHT. The question thus remains: do transgender individuals retain susceptibility to autoimmune disorders, malignancies, and infection from their sex assigned at birth? Or do they adopt the disease susceptibility of their transitioned gender through hormonal influences? Alternatively, might there be a “middle ground”, where some disease risks remain unchanged and others are increased or decreased upon transitioning? Therefore, transgender individuals present an opportunity to explore the influence of genetics vs hormones on sexual dimorphism in immunity. These questions can also be addressed with the Four Core Genotypes mouse model (180, 181) of four different combinations of gonads and sex chromosomes. This model has been successfully used to identify sex chromosome influences on physical traits, such as obesity and food intake (182, 183), and it should be readily possible to study muscle function in the same way.

In humans there have been cases of amelioration of subacute cutaneous lupus in transgender males receiving exogenous testosterone (184), and development of SLE and lupus nephritis in transgender females receiving exogenous estrogen (185–187), suggesting that the sexual dimorphism in lupus may be dependent on female sex hormones (or the suppression of testosterone). However, literature in this area is sparse, and there are currently no longitudinal studies on the long-term effects of GAHT on the immune cell populations, epigenome, or immune-related disease risks. One study has shown changes in methylation levels at the promoters of hormone receptor genes during GAHT, with increased methylation of the *AR* in transgender people on feminizing hormones (at 12 months of hormone therapy) and increased methylation of *ESR1* in transgender people on masculinizing hormones (at 6 and 12 months of hormone therapy) (188). Additionally, gene expression of *AR* was significantly reduced in transgender people on masculinizing hormone therapy (188). Methylation of hormone receptor genes was correlated with a variety of metabolic, anthropometric, inflammatory, and hormonal measures, including white blood cell counts, C-reactive protein, and cholesterol levels (188). Changes in the transcriptome have also been observed, with one small cross-sectional study reporting a unique transcriptome of rectal mucosal cells in transgender women compared to cis-males (189). Gene set enrichment analysis revealed enriched immunological pathway signatures which may have an impact on anally transmitted HIV infection (189).

## Importance of Sexual Dimorphism in Innate Immunity—Outstanding Questions

**Figure 3:**
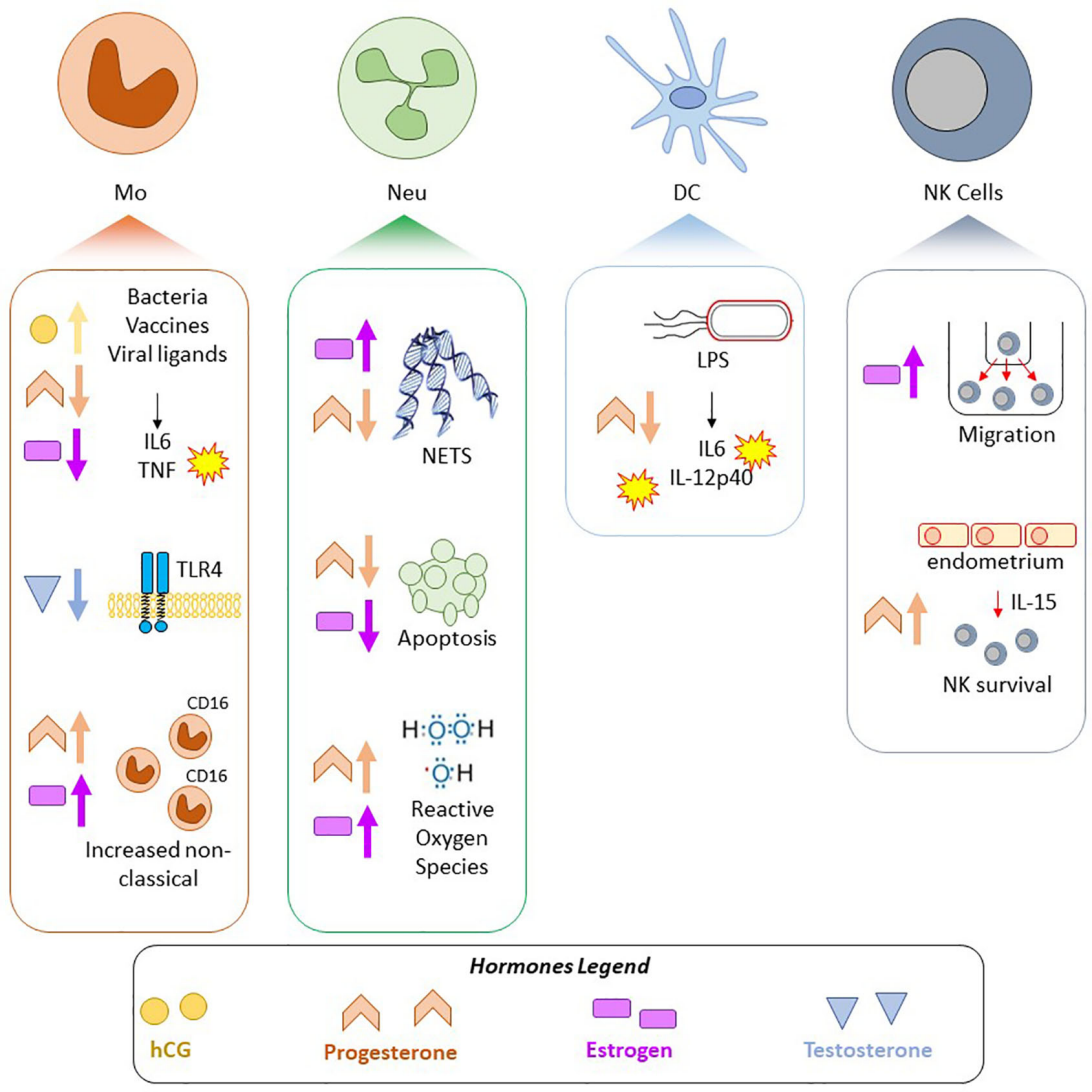
Summary of selected pregnancy-associated and sex hormone effects on innate immune cells. Findings in four cell types, Mo, monocytes/macrophages; Neu, Neutrophils; DC, Dendritic cells; NK, Natural Killer cells; are shown. Shapes correspond to hormone: yellow circle—hCG, pink rectangle—estrogen, orange chevron—progesterone, blue triangle—testosterone, and arrows indicate if the hormone attenuated or increased a response/phenotype.

Emerging evidence indicates that periods of profound hormonal shifts (including puberty, pregnancy, menopause, MHT, and GAHT) elicit a broad range of immunological, transcriptional, or epigenetic adaptations in blood or immune cells (**Figure 3**). In addition, there is evidence for a sex-specific transcriptome and methylome in circulating immune cells of adult males and females. The effects of sex hormones on immune cells, whether it be *via* classical nuclear hormone receptor signaling, non-classical hormone signaling, or *via* downstream epigenetic remodeling, are potential drivers of the sexually dimorphic aspects of immunity. In the era of personalized medicine, sex and gender are important factors to consider to ensure safe and effective treatment. This is of particular importance in the context of drugs targeting the immune system, as sex-specific differences in immune function and immune-reactogenicity have been well-demonstrated (190, 191).

Looking ahead, we propose that *in vitro* studies of human innate immune cells be performed to better understand the effects of particular hormones on their transcriptome, methylome, chromatin landscape, and immune function, and that these be done in conjunction with longitudinal, multi-epigenomic studies of human innate immune cells across puberty, pregnancy, menopause, MHT, and GAHT, since doing so will ultimately contribute to our understanding of infection risk and autoimmunity.

## Author Contributions

RSh and BN wrote the draft manuscript and made figures. AC, KP, and RSa edited the manuscript and added references. All authors contributed to the article and approved the submitted version.

## Funding

BN was supported by an NHMRC (Australia) Investigator Grant (no. 1173314) and Project Grant (no. 1157556).

## Conflict of Interest

The authors declare that the research was conducted in the absence of any commercial or financial relationships that could be construed as a potential conflict of interest.

## References

[1] Chaplin DD Overview of the immune response. J Allergy Clin Immunol (2010) 125(2 Suppl 2):S3–S23. 10.1016/j.jaci.2009.12.980 20176265 PMC2923430

[2] de Maat MP Bladbjerg EM Hjelmborg J Bathum L Jespersen J Christensen K Genetic influence on inflammation variables in the elderly. Arterioscler Thromb Vasc Biol (2004) 24(11):2168–73. 10.1161/01.ATV.0000143856.01669.e7 15345506

[3] Wessel J Moratorio G Rao F Mahata M Zhang L Greene W C-reactive protein, an ‘intermediate phenotype’ for inflammation: human twin studies reveal heritability, association with blood pressure and the metabolic syndrome, and the influence of common polymorphism at catecholaminergic/beta-adrenergic pathway loci. J Hypertens (2007) 25(2):329–43. 10.1097/hjh.0b013e328011753e 17211240

[4] Netea MG Joosten LA Latz E Mills KH Natoli G Stunnenberg HG Trained immunity: A program of innate immune memory in health and disease. Science (2016) 352(6284):aaf1098. 10.1126/science.aaf1098 27102489 PMC5087274

[5] Netea MG Dominguez-Andres J Barreiro LB Chavakis T Divangahi M Fuchs E Defining trained immunity and its role in health and disease. Nat Rev Immunol (2020) 20(6):375–88. 10.1038/s41577-020-0285-6 PMC7186935 32132681

[6] Kleinnijenhuis J Quintin J Preijers F Joosten LA Ifrim DC Saeed S Bacille Calmette-Guerin induces NOD2-dependent nonspecific protection from reinfection via epigenetic reprogramming of monocytes. Proc Natl Acad Sci U S A (2012) 109(43):17537–42. 10.1073/pnas.1202870109 PMC3491454 22988082

[7] Quintin J Saeed S Martens JHA Giamarellos-Bourboulis EJ Ifrim DC Logie C Candida albicans infection affords protection against reinfection via functional reprogramming of monocytes. Cell Host Microbe (2012) 12(2):223–32. 10.1016/j.chom.2012.06.006 PMC3864037 22901542

[8] Arts RJ Novakovic B Ter Horst R Carvalho A Bekkering S Lachmandas E Glutaminolysis and Fumarate Accumulation Integrate Immunometabolic and Epigenetic Programs in Trained Immunity. Cell Metab (2016) 24(6):807–19. 10.1016/j.cmet.2016.10.008 PMC5742541 27866838

[9] Bekkering S Arts RJW Novakovic B Kourtzelis I van der Heijden C Li Y Metabolic Induction of Trained Immunity through the Mevalonate Pathway. Cell (2018) 172(1-2):135–146 e9. 10.1016/j.cell.2017.11.025 29328908

[10] Jentho E Novakovic B Ruiz-Moreno C Kourtzelis I Martins R Chavakis T Heme induces innate immune memory. bioRxiv (2019) 2019.12.12.874578. 10.1101/2019.12.12.874578

[11] Ter Horst R Jaeger M Smeekens SP Oosting M Swertz MA Li Y Host and Environmental Factors Influencing Individual Human Cytokine Responses. Cell (2016) 167(4):1111–24.e13. 10.1016/j.cell.2016.10.018 27814508 PMC5787854

[12] Ibrahim A Morais S Ferro A Lunet N Peleteiro B Sex-differences in the prevalence of Helicobacter pylori infection in pediatric and adult populations: Systematic review and meta-analysis of 244 studies. Digest Liver Dis (2017) 49(7):742–9. 10.1016/j.dld.2017.03.019 28495503

[13] Neyrolles O Quintana-Murci L Sexual inequality in tuberculosis. PLoS Med (2009) 6(12):e1000199–e1000199. 10.1371/journal.pmed.1000199 20027210 PMC2788129

[14] Baig S Gender disparity in infections of Hepatitis B virus. J Coll Phys Surge–Pakistan: JCPSP (2009) 19(9):598–600. 09.2009/JCPSP.598600 19728952

[15] Jaillon S Berthenet K Garlanda C Sexual Dimorphism in Innate Immunity. Clin Rev Allergy Immunol (2019) 56(3):308–21. 10.1007/s12016-017-8648-x 28963611

[16] Williamson EJ Walker AJ Bhaskaran K Bacon S Bates C Morton CE OpenSAFELY: factors associated with COVID-19 death in 17 million patients. Nature (2020) 584(7821):430–6. 10.1038/s41586-020-2521-4 PMC7611074 32640463

[17] Klein SL Jedlicka A Pekosz A The Xs and Y of immune responses to viral vaccines. Lancet Infect Dis (2010) 10(5):338–49. 10.1016/S1473-3099(10)70049-9 PMC6467501 20417416

[18] Aghaeepour N Ganio EA McIlwain D Tsai AS Tingle M Van Gassen S An immune clock of human pregnancy. Sci Immunol (2017) 2(15). 10.1126/sciimmunol.aan2946 PMC5701281 28864494

[19] Gamliel M Goldman-Wohl D Isaacson B Gur C Stein N Yamin R Trained Memory of Human Uterine NK Cells Enhances Their Function in Subsequent Pregnancies. Immunity (2018) 48(5):951–62.e5. 10.1016/j.immuni.2018.03.030 29768178

[20] Bouman A Heineman MJ Faas MM Sex hormones and the immune response in humans. Hum Reprod Update (2005) 11(4):411–23. 10.1093/humupd/dmi008 15817524

[21] Sever R Glass CK Signaling by nuclear receptors. Cold Spring Harbor Perspect Biol (2013) 5(3):a016709–a016709. 10.1101/cshperspect.a016709 PMC3578364 23457262

[22] Schwartz N Verma A Schwartz CB Schwartz Z Boyan BD Rapid steroid hormone actions via membrane receptors. Biochim Biophys Acta (BBA) - Mol Cell Res (2016) 1863(9):2289–98. 10.1016/j.bbamcr.2016.06.004 27288742

[23] Kovats S Carreras E Agrawal H “Sex steroid receptors in immune cells”. In: Sex hormones and immunity to infection. Berlin, Heidelberg: Springer (2010). p. 53–91.

[24] Phiel KL Henderson RA Adelman SJ Elloso MM Differential estrogen receptor gene expression in human peripheral blood mononuclear cell populations. Immunol Lett (2005) 97(1):107–13. 10.1016/j.imlet.2004.10.007 15626482

[25] Laffont S Rouquié N Azar P Seillet C Plumas J Aspord C X-chromosome complement and estrogen receptor signaling independently contribute to the enhanced TLR7-mediated IFN-α production of plasmacytoid dendritic cells from women. J Immunol (2014) 193(11):5444–52. 10.4049/jimmunol.1303400 25339659

[26] Seillet C Laffont S Trémollières F Rouquié N Ribot C Arnal J-F The TLR-mediated response of plasmacytoid dendritic cells is positively regulated by estradiol in vivo through cell-intrinsic estrogen receptor α signaling. Blood (2012) 119(2):454–64. 10.1182/blood-2011-08-371831 22096248

[27] Driggers PH Segars JH Estrogen action and cytoplasmic signaling pathways. Part II: the role of growth factors and phosphorylation in estrogen signaling. Trends Endocrinol Metab: TEM (2002) 13(10):422–7. 10.1016/S1043-2760(02)00634-3 PMC4152897 12431838

[28] Kovats S Estrogen receptors regulate innate immune cells and signaling pathways. Cell Immunol (2015) 294(2):63–9. 10.1016/j.cellimm.2015.01.018 PMC4380804 25682174

[29] Khan D Ansar Ahmed S The Immune System Is a Natural Target for Estrogen Action: Opposing Effects of Estrogen in Two Prototypical Autoimmune Diseases. Front Immunol (2016) 6(635):1–8. 10.3389/fimmu.2015.00635 PMC4701921 26779182

[30] Chadwick CC Chippari S Matelan E Borges-Marcucci L Eckert AM Keith JC Identification of pathway-selective estrogen receptor ligands that inhibit NF-κB transcriptional activity. Proc Natl Acad Sci (2005) 102(7):2543–8. 10.1073/pnas.0405841102 PMC548967 15699342

[31] Demyanets S Pfaffenberger S Kaun C Rega G Speidl WS Kastl SP The estrogen metabolite 17β-dihydroequilenin counteracts interleukin-1α induced expression of inflammatory mediators in human endothelial cells in vitro via NF-κB pathway. Thromb Haemostasis (2006) 95(01):107–16. 10.1160/TH05-05-0333 16543969

[32] Evans MJ Eckert A Lai K Adelman SJ Harnish DC Reciprocal antagonism between estrogen receptor and NF-κB activity in vivo. Circ Res (2001) 89(9):823–30. 10.1161/hh2101.098543 11679413

[33] Nettles KW Gil G Nowak J Métivier R Sharma VB Greene GL CBP Is a dosage-dependent regulator of nuclear factor-kappaB suppression by the estrogen receptor. Mol Endocrinol (Baltimore Md) (2008) 22(2):263–72. 10.1210/me.2007-0324 PMC2234588 17932106

[34] Pelekanou V Kampa M Kiagiadaki F Deli A Theodoropoulos P Agrogiannis G Estrogen anti-inflammatory activity on human monocytes is mediated through cross-talk between estrogen receptor ERα36 and GPR30/GPER1. J Leukocyte Biol (2016) 99(2):333–47. 10.1189/jlb.3A0914-430RR 26394816

[35] Liu T Zhang L Joo D Sun SC NF-kappaB signaling in inflammation. Signal Transduct Target Ther (2017) 2. 10.1038/sigtrans.2017.23 PMC5661633 29158945

[36] Miraghazadeh B Cook MC Nuclear Factor-kappaB in Autoimmunity: Man and Mouse. Front Immunol (2018) 9:613. 10.3389/fimmu.2018.00613 29686669 PMC5900062

[37] Linowiecka K Urbanowska-Domańska O Guz J Foksiński M The potential influence of breast cancer estrogen receptors’ distribution on active DNA demethylation. Contemp Oncol (Poznan Poland) (2019) 23(2):74–80. 10.5114/wo.2019.85200 PMC6630393 31316288

[38] Kasubuchi M Watanabe K Hirano K Inoue D Li X Terasawa K Membrane progesterone receptor beta (mPRβ/Paqr8) promotes progesterone-dependent neurite outgrowth in PC12 neuronal cells via non-G protein-coupled receptor (GPCR) signaling. Sci Rep (2017) 7(1):5168. 10.1038/s41598-017-05423-9 28701790 PMC5507890

[39] Khan KN Masuzaki H Fujishita A Kitajima M Sekine I Matsuyama T Estrogen and progesterone receptor expression in macrophages and regulation of hepatocyte growth factor by ovarian steroids in women with endometriosis. Hum Reprod (2005) 20(7):2004–13. 10.1093/humrep/deh897 15831511

[40] Hall OJ Klein SL Progesterone-based compounds affect immune responses and susceptibility to infections at diverse mucosal sites. Mucosal Immunol (2017) 10(5):1097–107. 10.1038/mi.2017.35 28401937

[41] Butts CL Bowers E Horn JC Shukair SA Belyavskaya E Tonelli L Inhibitory effects of progesterone differ in dendritic cells from female and male rodents. Gender Med (2008) 5(4):434–47. 10.1016/j.genm.2008.11.001 PMC2941400 19108816

[42] Robinson DP Klein SL Pregnancy and pregnancy-associated hormones alter immune responses and disease pathogenesis. Hormones Behav (2012) 62(3):263–71. 10.1016/j.yhbeh.2012.02.023 PMC3376705 22406114

[43] Jones LA Kreem S Shweash M Paul A Alexander J Roberts CW Differential Modulation of TLR3- and TLR4-Mediated Dendritic Cell Maturation and Function by Progesterone. J Immunol (2010) 185(8):4525. 10.4049/jimmunol.0901155 20844199

[44] Jones LA Anthony J-P Henriquez FL Lyons RE Nickdel MB Carter KC Toll-like receptor-4-mediated macrophage activation is differentially regulated by progesterone via the glucocorticoid and progesterone receptors. Immunology (2008) 125(1):59–69. 10.1111/j.1365-2567.2008.02820.x 18373668 PMC2526260

[45] Davey RA Grossmann M Androgen Receptor Structure, Function and Biology: From Bench to Bedside. Clin Biochem Rev (2016) 37(1):3–15. 27057074 PMC4810760

[46] Gubbels Bupp MR Jorgensen TN Androgen-Induced Immunosuppression. Front Immunol (2018) 9:794–4. 10.3389/fimmu.2018.00794 PMC5932344 29755457

[47] Pergola C Rogge A Dodt G Northoff H Weinigel C Barz D Testosterone suppresses phospholipase D, causing sex differences in leukotriene biosynthesis in human monocytes. FASEB J (2011) 25(10):3377–87. 10.1096/fj.11-182758 21693622

[48] Capellino S Villaggio V Montagna P Sulli A Craviotto C Cutolo M [17beta-Estradiol and testosterone influence the mRNA expression and the time course of inflammatory cytokines in activated human monocytic cell line (THP-1)]. Reumatismo (2005) 57(3):193–6. 10.4081/reumatismo.2005.193 16258604

[49] Rettew JA Huet-Hudson YM Marriott I Testosterone Reduces Macrophage Expression in the Mouse of Toll-Like Receptor 4, a Trigger for Inflammation and Innate Immunity. Biol Reprod (2008) 78(3):432–7. 10.1095/biolreprod.107.063545 18003947

[50] Posma E Moes H Heineman MJ Faas MM The effect of testosterone on cytokine production in the specific and non-specific immune response. Am J Reprod Immunol (New York NY: 1989) (2004) 52(4):237–43. 10.1111/j.1600-0897.2004.00216.x 15494044

[51] de Bree CLCJ Janssen R Aaby P van Crevel R Joosten LAB Benn CS The impact of sex hormones on BCG-induced trained immunity. J Leukocyte Biol (2018) 104(3):573. 10.1002/JLB.5MA0118-027R 30153369

[52] Veldhuijzen DS Keaser ML Traub DS Zhuo J Gullapalli RP Greenspan JD The role of circulating sex hormones in menstrual cycle-dependent modulation of pain-related brain activation. Pain (2013) 154(4):548–59. 10.1016/j.pain.2012.12.019 PMC3608932 23528204

[53] Straub RH The complex role of estrogens in inflammation. Endocr Rev (2007) 28(5):521–74. 10.1210/er.2007-0001 17640948

[54] Deshpande R Khalili H Pergolizzi RG Michael SD Chang MDY Estradiol down-regulates LPS-induced cytokine production and NFkB activation in murine macrophages. Am J Reprod Immunol (1997) 38(1):46–54. 10.1111/j.1600-0897.1997.tb00275.x 9266010

[55] Polan ML Daniele A Kuo A Gonadal steroids modulate human monocyte interleukin-1 (IL-1) activity. Fertil Steril (1988) 49(6):964–8. 10.1016/S0015-0282(16)59945-2 2967196

[56] Shanker G Sorci-Thomas M Register T Adams M The inducible expression of THP-1 cell interleukin-1 mRNA: Effects of estrogen on differential response to phorbol ester and lipopolysaccharide. Lymphokine Cytokine Res (1994) 13:1–7. 8186319

[57] Asai K Hiki N Mimura Y Ogawa T Unou K Kaminishi M Gender Differences in Cytokine Secretion by Human Peripheral Blood Mononuclear Cells: Role of Estrogen in Modulating LPS-Induced Cytokine Secretion in an Ex Vivo Septic Model. United States: BIOMEDICAL PRESS (2001). p. 340. 10.1097/00024382-200116050-00003 11699070

[58] Kaplan MJ Radic M Neutrophil extracellular traps: double-edged swords of innate immunity. J Immunol (Baltimore Md 1950) (2012) 189(6):2689–95. 10.4049/jimmunol.1201719 PMC3439169 22956760

[59] Molloy EJ O’Neill AJ Grantham JJ Sheridan-Pereira M Fitzpatrick JM Webb DW Sex-specific alterations in neutrophil apoptosis: the role of estradiol and progesterone. Blood (2003) 102(7):2653–9. 10.1182/blood-2003-02-0649 12791649

[60] Miller AP Feng W Xing D Weathington NM Blalock JE Chen Y-F Estrogen Modulates Inflammatory Mediator Expression and Neutrophil Chemotaxis in Injured Arteries. Circulation (2004) 110(12):1664–9. 10.1161/01.CIR.0000142050.19488.C7 15353495

[61] Rettew JA Huet YM Marriott I Estrogens Augment Cell Surface TLR4 Expression on Murine Macrophages and Regulate Sepsis Susceptibility in Vivo. Endocrinology (2009) 150(8):3877–84. 10.1210/en.2009-0098 19406943

[62] Vázquez-Martínez ER García-Gómez E Camacho-Arroyo I González-Pedrajo B Sexual dimorphism in bacterial infections. Biol sex Dif (2018) 9(1):27–7. 10.1186/s13293-018-0187-5 PMC6011518 29925409

[63] Xu J Tong L Yao J Guo Z Lui KY Hu X Association of Sex With Clinical Outcome in Critically Ill Sepsis Patients: A Retrospective Analysis of the Large Clinical Database MIMIC-III. Shock (2019) 52(2). 10.1097/SHK.0000000000001253 PMC6687414 30138298

[64] Shah NM Lai PF Imami N Johnson MR Progesterone-Related Immune Modulation of Pregnancy and Labor. Front Endocrinol (2019) 10(198). 10.3389/fendo.2019.00198 PMC6449726 30984115

[65] Giaglis S Stoikou M Sur Chowdhury C Schaefer G Grimolizzi F Rossi SW Multimodal Regulation of NET Formation in Pregnancy: Progesterone Antagonizes the Pro-NETotic Effect of Estrogen and G-CSF. Front Immunol (2016) 7:565–5. 10.3389/fimmu.2016.00565 PMC5136684 27994595

[66] Menzies FM Henriquez FL Alexander J Roberts CW Selective inhibition and augmentation of alternative macrophage activation by progesterone. Immunology (2011) 134(3):281–91. 10.1111/j.1365-2567.2011.03488.x PMC3209568 21977998

[67] Pergola C Pergola C Schaible AM Nikels F Dodt G Northoff H Progesterone rapidly down-regulates the biosynthesis of 5-lipoxygenase products in human primary monocytes. Pharmacol Res (2015) 94:42–50. 10.1016/j.phrs.2015.01.007 25681061

[68] Ma W-T Gao F Gu K Chen D-K The Role of Monocytes and Macrophages in Autoimmune Diseases: A Comprehensive Review. Front Immunol (2019) 10:1140–0. 10.3389/fimmu.2019.01140 PMC6543461 31178867

[69] Gordon S Plüddemann A “Chapter 11 - Role of Macrophages in Autoimmunity”. In: Rose NR Mackay IR, editors. The Autoimmune Diseases, Fifth Edition Boston: Academic Press (2014). p. 161–74.

[70] Rubtsova K Marrack P Rubtsov AV Sexual dimorphism in autoimmunity. J Clin Invest (2015) 7):2187. 10.1172/JCI78082 PMC4497744 25915581

[71] Jacobson DL Gange SJ Rose NR Graham NM Epidemiology and estimated population burden of selected autoimmune diseases in the United States. Clin Immunol Immunopathol (1997) 84(3):223–43. 10.1006/clin.1997.4412 9281381

[72] Desai MK Brinton RD Autoimmune Disease in Women: Endocrine Transition and Risk Across the Lifespan. Front Endocrinol (2019) 10:265–5. 10.3389/fendo.2019.00265 PMC6501433 31110493

[73] Amador-Patarroyo MJ Rodriguez-Rodriguez A Montoya-Ortiz G How does age at onset influence the outcome of autoimmune diseases? Autoimmune Dis (2012) 2012:251730–0. 10.1155/2012/251730 PMC3238350 22195277

[74] Bove R Autoimmune diseases and reproductive aging. Clin Immunol (Orlando Fla) (2013) 149(2):251–64. 10.1016/j.clim.2013.02.010 PMC3805815 23522436

[75] Bynoté KK Hackenberg JM Korach KS Lubahn DB Lane PH Gould KA Estrogen receptor-α deficiency attenuates autoimmune disease in (NZB × NZW)F1 mice. Genes Immun (2008) 9(2):137–52. 10.1038/sj.gene.6364458 18200028

[76] Dulos J Vijn P van Doorn C Hofstra CL Veening-Griffioen D de Graaf J Suppression of the inflammatory response in experimental arthritis is mediated via estrogen receptor alpha but not estrogen receptor beta. Arthritis Res Ther (2010) 12(3):R101–1. 10.1186/ar3032 PMC2911889 20497523

[77] Morales LBJ Loo KK Liu H-b Peterson C Tiwari-Woodruff S Voskuhl RR Treatment with an Estrogen Receptor α Ligand Is Neuroprotective in Experimental Autoimmune Encephalomyelitis. J Neurosci (2006) 26(25):6823–33. 10.1523/JNEUROSCI.0453-06.2006 PMC6673842 16793889

[78] Bianchi VE The Anti-Inflammatory Effects of Testosterone. J Endocr Soc (2018) 3(1):91–107. 10.1210/js.2018-00186 30582096 PMC6299269

[79] Roubinian J Talal N Greenspan J Goodman J Siiteri P Effect of castration and sex hormone treatment on survival, anti-nucleic acid antibodies, and glomerulonephritis in NZB/NZW F1 mice. J Exp Med (1978) 147(6):1568–83. 10.1084/jem.147.6.1568 PMC2184317 308087

[80] Makino S Kunimoto K Muraoka Y Katagiri K Effect of Castration on the Appearance of Diabetes in NOD Mouse. Exp Anim (1981) 30(2):137–40. 10.1538/expanim1978.30.2_137 7286067

[81] Harbuz MS Perveen-Gill Z Lightman SL Jessop DS A Protective Role For Testosterone In Adjuvant-Induced Arthritis. Rheumatology (1995) 34(12):1117–22. 10.1093/rheumatology/34.12.1117 8608351

[82] Kanda N Tsuchida T Tamaki K Testosterone inhibits immunoglobulin production by human peripheral blood mononuclear cells. Clin Exp Immunol (1996) 106(2):410–5. 10.1046/j.1365-2249.1996.d01-842.x PMC2200579 8918592

[83] Kanda N Tsuchida T Tamaki K Testosterone suppresses anti-DNA antibody production in peripheral blood mononuclear cells from patients with systemic lupus erythematosus. Arthritis And Rheum (1997) 40(9):1703–11. 10.1002/art.1780400921 9324026

[84] Gordon D Beastall GH Thomson JA Sturrock RD Prolonged hypogonadism in male patients with rheumatoid arthritis during flares in disease activity. Br J Rheumatol (1988) 27(6):440–4. 10.1093/rheumatology/27.6.440 3144408

[85] Cutolo M Balleari E Giusti M Monachesi M Accardo S Sex hormone status of male patients with rheumatoid arthritis: evidence of low serum concentrations of testosterone at baseline and after human chorionic gonadotropin stimulation. Arthritis Rheum (1988) 31(10):1314–7. 10.1002/art.1780311015 3140823

[86] Spector TD Ollier W Perry LA Silman AJ Thompson PW Edwards A Free and serum testosterone levels in 276 males: a comparative study of rheumatoid arthritis, ankylosing spondylitis and healthy controls. Clin Rheumatol (1989) 8(1):37–41. 10.1007/BF02031066 2787224

[87] Martens HF Sheets PK Tenover JS Dugowson CE Bremner WJ Starkebaum G Decreased testosterone levels in men with rheumatoid arthritis: effect of low dose prednisone therapy. J Rheumatol (1994) 21(8):1427–31. 7983641

[88] Masi AT Feigenbaum SL Chatterton RT Hormonal and pregnancy relationships to rheumatoid arthritis: convergent effects with immunologic and microvascular systems. Semin Arthritis Rheum (1995) 25(1):1–27. 10.1016/S0049-0172(95)80014-X 8525387

[89] Kanik KS Chrousos GP Schumacher HR Crane ML Yarboro CH Wilder RL Adrenocorticotropin, glucocorticoid, and androgen secretion in patients with new onset synovitis/rheumatoid arthritis: relations with indices of inflammation. J Clin Endocrinol Metab (2000) 85(4):1461–6. 10.1210/jc.85.4.1461 10770182

[90] Tengstrand B Carlström K Hafström I Gonadal hormones in men with rheumatoid arthritis–from onset through 2 years. J Rheumatol (2009) 36(5):887–92. 10.3899/jrheum.080558 19273455

[91] Tengstrand B Carlström K Hafström I Bioavailable testosterone in men with rheumatoid arthritis-high frequency of hypogonadism. Rheumatol (Oxford) (2002) 41(3):285–9. 10.1093/rheumatology/41.3.285 11934965

[92] Jiménez-Balderas FJ Tápia-Serrano R Fonseca ME Arellano J Beltrán A Yáñez A High frequency of association of rheumatic/autoimmune diseases and untreated male hypogonadism with severe testicular dysfunction. Arthritis Res (2001) 3(6):362–7. 10.1186/ar328 PMC64847 11714390

[93] Inman RD Jovanovic L Markenson JA Longcope C Dawood MY Lockshin MD Systemic lupus erythematosus in men. Genetic and endocrine features. Arch Intern Med (1982) 142(10):1813–5. 10.1001/archinte.142.10.1813 7125767

[94] Carrabba M Giovine C Chevallard M Angelini M Ambrosi B Travaglini P Abnormalities of sex hormones in men with systemic lupus erythematosus. Clin Rheumatol (1985) 4(4):420–5. 10.1007/BF02031894 3830519

[95] Folomeev M Dougados M Beaune J Kouyoumdjian JC Nahoul K Amor B Plasma sex hormones and aromatase activity in tissues of patients with systemic lupus erythematosus. Lupus (1992) 1(3):191–5. 10.1177/096120339200100312 1301981

[96] Sequeira JF Keser G Greenstein B Wheeler MJ Duarte PC Khamashta MA Systemic lupus erythematosus: sex hormones in male patients. Lupus (1993) 2(5):315–7. 10.1177/096120339300200507 8305925

[97] Vilarinho ST Costallat LT Evaluation of the hypothalamic-pituitary-gonadal axis in males with systemic lupus erythematosus. J Rheumatol (1998) 25(6):1097–103. 9632070

[98] Chang DM Chang CC Kuo SY Chu SJ Chang ML Hormonal profiles and immunological studies of male lupus in Taiwan. Clin Rheumatol (1999) 18(2):158–62. 10.1007/s100670050075 10357123

[99] Mok CC Lau CS Profile of sex hormones in male patients with systemic lupus erythematosus. Lupus (2000) 9(4):252–7. 10.1191/096120300680198926 10866095

[100] Bhattoa HP Kiss E Bettembuk P Balogh A Bone mineral density, biochemical markers of bone turnover, and hormonal status in men with systemic lupus erythematosus. Rheumatol Int (2001) 21(3):97–102. 10.1007/s00296-001-0149-8 11765229

[101] Vecchi AP Borba EF Bonfá E Cocuzza M Pieri P Kim CA Penile anthropometry in systemic lupus erythematosus patients. Lupus (2011) 20(5):512–8. 10.1177/0961203310384121 21282296

[102] Wei T Lightman SL The neuroendocrine axis in patients with multiple sclerosis. Brain (1997) 120( Pt 6):1067–76. 10.1093/brain/120.6.1067 9217689

[103] Safarinejad MR Evaluation of endocrine profile, hypothalamic-pituitary-testis axis and semen quality in multiple sclerosis. J Neuroendocrinol (2008) 20(12):1368–75. 10.1111/j.1365-2826.2008.01791.x 19094084

[104] Baillargeon J Al Snih S Raji MA Urban RJ Sharma G Sheffield-Moore M Hypogonadism and the risk of rheumatic autoimmune disease. Clin Rheumatol (2016) 35(12):2983–7. 10.1007/s10067-016-3330-x PMC5544431 27325124

[105] Chen Y Chen Y Xia F Wang N Chen C Nie X A Higher Ratio of Estradiol to Testosterone Is Associated with Autoimmune Thyroid Disease in Males. Thyroid (2017) 27(7):960–6. 10.1089/thy.2016.0661 28558486

[106] La-or C Wichai A Boonsong O The relationship between circulating estradiol and thyroid autoimmunity in males. Eur J Endocrinol (2014) 170(1):63–7. 10.1530/EJE-13-0455 24128431

[107] Baker Frost D Wolf B Peoples C Fike J Silver K Laffoon M Estradiol levels are elevated in older men with diffuse cutaneous SSc and are associated with decreased survival. Arthritis Res Ther (2019) 21(1):85. 10.1186/s13075-019-1870-6 30940202 PMC6444502

[108] Bongen E Lucian H Khatri A Fragiadakis GK Bjornson ZB Nolan GP Sex Differences in the Blood Transcriptome Identify Robust Changes in Immune Cell Proportions with Aging and Influenza Infection. Cell Rep (2019) 29(7):1961–73.e4. 10.1016/j.celrep.2019.10.019 31722210 PMC6856718

[109] Jansen R Batista S Brooks AI Tischfield JA Willemsen G van Grootheest G Sex differences in the human peripheral blood transcriptome. BMC Genomics (2014) 15(1):33. 10.1186/1471-2164-15-33 24438232 PMC3904696

[110] Gal-Oz ST Maier B Yoshida H Seddu K Elbaz N Czysz C ImmGen report: sexual dimorphism in the immune system transcriptome. Nat Commun (2019) 10(1):4295–5. 10.1038/s41467-019-12348-6 PMC6754408 31541153

[111] Mamrut S Avidan N Staun-Ram E Ginzburg E Truffault F Berrih-Aknin S Integrative analysis of methylome and transcriptome in human blood identifies extensive sex-and immune cell-specific differentially methylated regions. Epigenetics (2015) 10(10):943–57. 10.1080/15592294.2015.1084462 PMC4844205 26291385

[112] Natoli G Ostuni R Adaptation and memory in immune responses. Nat Immunol (2019) 20(7):783–92. 10.1038/s41590-019-0399-9 31213714

[113] Bird A Perceptions of epigenetics. Nature (2007) 447(7143):396–8. 10.1038/nature05913 17522671

[114] Ostuni R Piccolo V Barozzi I Polletti S Termanini A Bonifacio S Latent enhancers activated by stimulation in differentiated cells. Cell (2013) 152(1-2):157–71. 10.1016/j.cell.2012.12.018 23332752

[115] Kuznetsova T Prange KHM Glass CK de Winther MPJ Transcriptional and epigenetic regulation of macrophages in atherosclerosis. Nat Rev Cardiol (2020) 17(4):216–28. 10.1038/s41569-019-0265-3 PMC7770754 31578516

[116] Novakovic B Habibi E Wang SY Arts RJW Davar R Megchelenbrink W beta-Glucan Reverses the Epigenetic State of LPS-Induced Immunological Tolerance. Cell (2016) 167(5):1354–68.e14. 10.1016/j.cell.2016.09.034 27863248 PMC5927328

[117] Saeed S Quintin J Kerstens HH Rao NA Aghajanirefah A Matarese F Epigenetic programming of monocyte-to-macrophage differentiation and trained innate immunity. Science (2014) 345(6204):1251086. 10.1126/science.1251086 25258085 PMC4242194

[118] Dekkers KF Neele AE Jukema JW Heijmans BT de Winther MPJ Human monocyte-to-macrophage differentiation involves highly localized gain and loss of DNA methylation at transcription factor binding sites. Epigenet Chromatin (2019) 12(1):34. 10.1186/s13072-019-0279-4 PMC6551876 31171035

[119] Wang C Nanni L Novakovic B Megchelenbrink W Kuznetsova T Stunnenberg HG Extensive epigenomic integration of the glucocorticoid response in primary human monocytes and in vitro derived macrophages. Sci Rep (2019) 9(1):2772. 10.1038/s41598-019-39395-9 30809020 PMC6391480

[120] Mann M Cortez V Vadlamudi RK Epigenetics of estrogen receptor signaling: role in hormonal cancer progression and therapy. Cancers (2011) 3(3):1691–707. 10.3390/cancers3021691 PMC3147309 21814622

[121] Houshdaran S Oke AB Fung JC Vo KC Nezhat C Giudice LC Steroid hormones regulate genome-wide epigenetic programming and gene transcription in human endometrial cells with marked aberrancies in endometriosis. PLoS Genet (2020) 16(6):e1008601. 10.1371/journal.pgen.1008601 32555663 PMC7299312

[122] Thompson EE Nicodemus-Johnson J Kim KW Gern JE Jackson DJ Lemanske RFC Global DNA methylation changes spanning puberty are near predicted estrogen-responsive genes and enriched for genes involved in endocrine and immune processes. Clin Epigenet (2018) 1). 10.1186/s13148-018-0491-2 PMC5941468 29760811

[123] Almstrup K Lindhardt Johansen M Busch AS Hagen CP Nielsen JE Petersen JH Pubertal development in healthy children is mirrored by DNA methylation patterns in peripheral blood. Sci Rep (2016) 6(1):28657. 10.1038/srep28657 27349168 PMC4923870

[124] Gruzieva O Merid SK Chen S Mukherjee N Hedman AM Almqvist C DNA Methylation Trajectories During Pregnancy. Epigenet Insights (2019) 12:2516865719867090. 10.1177/2516865719867090 31453433 PMC6696836

[125] Gomez-Lopez N Romero R Hassan SS Bhatti G Berry SM Kusanovic JP The Cellular Transcriptome in the Maternal Circulation During Normal Pregnancy: A Longitudinal Study. Front Immunol (2019) 10(2863). 10.3389/fimmu.2019.02863 PMC6928201 31921132

[126] Hong S Banchereau R Maslow BL Guerra MM Cardenas J Baisch J Longitudinal profiling of human blood transcriptome in healthy and lupus pregnancy. J Exp Med (2019) 216(5):1154–69. 10.1084/jem.20190185 PMC6504211 30962246

[127] Michalczyk AA Janus ED Judge A Ebeling PR Best JD Ackland MJ Transient epigenomic changes during pregnancy and early postpartum in women with and without type 2 diabetes. Epigenomics (2018) 10(4):419–31. 10.2217/epi-2017-0129 PMC5925439 29561170

[128] Dvornyk V Liu Y Lu Y Shen H Lappe JM Recker RR Effect of Menopause on Gene Expression Profiles of Circulating Monocytes: A Pilot in vivo Microarray Study. J Genet Genomics (2007) 34(11):974–83. 10.1016/S1673-8527(07)60110-6 18037134

[129] Bahl A Laakkonen E Ismail K Sipilä S Mikkola T Berglund E Hormone Replacement Therapy Associated White Blood Cell DNA Methylation and Gene Expression are Associated With Within-Pair Differences of Body Adiposity and Bone Mass. Twin Res Hum Genet (2015) 18:647–61. 10.1017/thg.2015.82 26678050

[130] Valiathan R Ashman M Asthana D Effects of Ageing on the Immune System: Infants to Elderly. Scand J Immunol (2016) 83(4):255–66. 10.1111/sji.12413 26808160

[131] Tollerud DJ Ildstad ST Brown LM Clark JW Blattner WA Mann DL T-cell subsets in healthy teenagers: Transition to the adult phenotype. Clin Immunol Immunopathol (1990) 56(1):88–96. 10.1016/0090-1229(90)90172-M 2357861

[132] Bartlett JA Schleifer SJ Demetrikopoulos MK Delaney BR Shiflett SC Keller SE Immune Function in Healthy Adolescents. Clin Diagn Lab Immunol (1998) 5(1):105. 10.1128/CDLI.5.1.105-113.1998 9455890 PMC121401

[133] Kumar P Magon N Hormones in pregnancy. Nigerian Med J J Nigeria Med Assoc (2012) 53(4):179–83. 10.4103/0300-1652.107549 PMC3640235 23661874

[134] Schumacher A Human Chorionic Gonadotropin as a Pivotal Endocrine Immune Regulator Initiating and Preserving Fetal Tolerance. Int J Mol Sci (2017) 18(10):2166. 10.3390/ijms18102166 PMC5666847 29039764

[135] Bansal AS Bora SA Saso S Smith JR Johnson MR Thum MY Mechanism of human chorionic gonadotrophin-mediated immunomodulation in pregnancy. Expert Rev Clin Immunol (2012) 8(8):747–53. 10.1586/eci.12.77 23167686

[136] Reinhard G Noll A Schlebusch H Mallmann P Ruecker AV Shifts in the TH1/TH2 Balance during Human Pregnancy Correlate with Apoptotic Changes. Biochem Biophys Res Commun (1998) 245(3):933–8. 10.1006/bbrc.1998.8549 9588218

[137] Sykes L MacIntyre DA Yap XJ Teoh TG Bennett PR The Th1:th2 dichotomy of pregnancy and preterm labour. Mediators Inflamm (2012) 2012:967629–9. 10.1155/2012/967629 PMC3376783 22719180

[138] Tranchot-Diallo J Gras G Benveniste O MarcÉ D Roques P Dormont D Modulations of Cytokine Expression in Pregnant Women. Am J Reprod Immunol (1997) 37(3):215–26. 10.1111/j.1600-0897.1997.tb00218.x 9127642

[139] Szekeres-Bartho J Šućurović S Mulac-Jeričević B The Role of Extracellular Vesicles and PIBF in Embryo-Maternal Immune-Interactions. Front Immunol (2018) 9(2890):1–9. 10.3389/fimmu.2018.02890 30619262 PMC6300489

[140] Marzi M Vigano A Trabattoni D Villa ML Salvaggio A Clerici E Characterization of type 1 and type 2 cytokine production profile in physiologic and pathologic human pregnancy. Great Britain: Blackwell Scientific Publishers (1996). p. 127. 10.1046/j.1365-2249.1996.d01-809.x PMC2200555 8870710

[141] Makhseed M Raghupathy R Azizieh F Omu A Al-Shamali E Ashkanani L Th1 and Th2 cytokine profiles in recurrent aborters with successful pregnancy and with subsequent abortions. Hum Reprod (2001) 16(10):2219–26. 10.1093/humrep/16.10.2219 11574519

[142] McCracken SA Gallery E Morris JM Pregnancy-specific down-regulation of NF-kappa B expression in T cells in humans is essential for the maintenance of the cytokine profile required for pregnancy success. J Immunol (2004) 172(7):4583–91. 10.4049/jimmunol.172.7.4583 15034076

[143] Liang PY Diao LH Huang CY Lian RC Chen X Li GG The pro-inflammatory and anti-inflammatory cytokine profile in peripheral blood of women with recurrent implantation failure. Reprod BioMed Online (2015) 31(6):823–6. 10.1016/j.rbmo.2015.08.009 26371706

[144] Aluvihare VR Kallikourdis M Betz AG Regulatory T cells mediate maternal tolerance to the fetus. Nat Immunol (2004) 5(3):266–71. 10.1038/ni1037 14758358

[145] Somerset DA Zheng Y Kilby MD Sansom DM Drayson MT Normal human pregnancy is associated with an elevation in the immune suppressive CD25+ CD4+ regulatory T-cell subset. Immunology (2004) 112(1):38–43. 10.1111/j.1365-2567.2004.01869.x 15096182 PMC1782465

[146] Shima T Sasaki Y Itoh M Nakashima A Ishii N Sugamura K Regulatory T cells are necessary for implantation and maintenance of early pregnancy but not late pregnancy in allogeneic mice. J Reprod Immunol (2010) 85(2):121–9. 10.1016/j.jri.2010.02.006 20439117

[147] Shirshev SV Orlova EG Zamorina SA Nekrasova IV Influence of reproductive hormones on the induction of CD4(+)CD25 (bright)Foxp (3+) regulatory T cells. Dokl Biol Sci (2011) 440:343–6. 10.1134/S0012496611050024 22134828

[148] Dauven D Ehrentraut S Langwisch S Zenclussen AC Schumacher A Immune Modulatory Effects of Human Chorionic Gonadotropin on Dendritic Cells Supporting Fetal Survival in Murine Pregnancy. Front Endocrinol (2016) 7(146). 10.3389/fendo.2016.00146 PMC5108759 27895621

[149] Schumacher A Brachwitz N Sohr S Engeland K Langwisch S Dolaptchieva M Human chorionic gonadotropin attracts regulatory T cells into the fetal-maternal interface during early human pregnancy. J Immunol (2009) 182(9):5488–97. 10.4049/jimmunol.0803177 19380797

[150] Silasi M You Y Simpson S Kaislasuo J Pal L Guller S Human Chorionic Gonadotropin modulates CXCL10 Expression through Histone Methylation in human decidua. Sci Rep (2020) 10(1):5785. 10.1038/s41598-020-62593-9 32238853 PMC7113245

[151] Nagamatsu T Schust DJ The contribution of macrophages to normal and pathological pregnancies. Am J Reprod Immunol (2010) 63(6):460–71. 10.1111/j.1600-0897.2010.00813.x 20163399

[152] Gustafsson C Mjösberg J Matussek A Geffers R Matthiesen L Berg G Gene Expression Profiling of Human Decidual Macrophages: Evidence for Immunosuppressive Phenotype. PLoS One (2008) 3(4):e2078. 10.1371/journal.pone.0002078 18446208 PMC2323105

[153] Pflitsch C Feldmann CN Richert L Hagen S Diemert A Goletzke J In-depth characterization of monocyte subsets during the course of healthy pregnancy. J Reprod Immunol (2020) 141:103151. 10.1016/j.jri.2020.103151 32531656

[154] Ziegler SM Feldmann CN Hagen SH Richert L Barkhausen T Goletzke J Innate immune responses to toll-like receptor stimulation are altered during the course of pregnancy. J Reprod Immunol (2018) 128:30–7. 10.1016/j.jri.2018.05.009 29886307

[155] Veenstra van Nieuwenhoven AL Bouman A Moes H Heineman MJ de Leij LFMH Santema J Endotoxin-induced cytokine production of monocytes of third-trimester pregnant women compared with women in the follicular phase of the menstrual cycle. Am J Obstetr Gynecol (2003) 188(4):1073–7. 10.1067/mob.2003.263 12712113

[156] Luppi P Haluszczak C Betters D Richard CAH Trucco M DeLoia JA Monocytes are progressively activated in the circulation of pregnant women. United States: BUSINESS AND REDACTORY SERVICES (2002). p. 874. 12429709

[157] Rami D La Bianca M Agostinis C Zauli G Radillo O Bulla R The first trimester gravid serum regulates procalcitonin expression in human macrophages skewing their phenotype in vitro. Mediators Inflamm (2014) 2014:248963–3. 10.1155/2014/248963 PMC3964843 24733960

[158] Gibson DA Greaves E Critchley HO Saunders PT Estrogen-dependent regulation of human uterine natural killer cells promotes vascular remodelling via secretion of CCL2. Hum Reprod (2015) 30(6):1290–301. 10.1093/humrep/dev067 PMC4498222 25820695

[159] Barber EM Pollard JW The uterine NK cell population requires IL-15 but these cells are not required for pregnancy nor the resolution of a Listeria monocytogenes infection. J Immunol (2003) 171(1):37–46. 10.4049/jimmunol.171.1.37 12816981

[160] Okada H Nakajima T Sanezumi M Ikuta A Yasuda K Kanzaki H Progesterone Enhances Interleukin-15 Production in Human Endometrial Stromal Cells in Vitro1. J Clin Endocrinol Metab (2000) 85(12):4765–70. 10.1210/jcem.85.12.7023 11134140

[161] Ostensen M Villiger PM The remission of rheumatoid arthritis during pregnancy. Semin In Immunopathol (2007) 29(2):185–91. 10.1007/s00281-007-0072-5 17621703

[162] Confavreux C Hutchinson M Hours MM Cortinovis-Tourniaire P Moreau T Rate of pregnancy-related relapse in multiple sclerosis. Pregnancy in Multiple Sclerosis Group. N Engl J Med (1998) 339(5):285–91. 10.1056/NEJM199807303390501 9682040

[163] Weetman AP Immunity, thyroid function and pregnancy: molecular mechanisms. Nat Rev Endocrinol (2010) 6(6):311–8. 10.1038/nrendo.2010.46 20421883

[164] Piccinni M-P Lombardelli L Logiodice F Kullolli O Parronchi P Romagnani S How pregnancy can affect autoimmune diseases progression? Clin Mol Allergy CMA (2016) 14:11–1. 10.1186/s12948-016-0048-x PMC5025626 27651750

[165] Lima F Buchanan NM Khamashta MA Kerslake S Hughes GR Obstetric outcome in systemic lupus erythematosus. Semin Arthritis Rheum (1995) 25(3):184–92. 10.1016/S0049-0172(95)80030-1 8650588

[166] Cortés-Hernández J Ordi-Ros J Paredes F Casellas M Castillo F Vilardell-Tarres M Clinical predictors of fetal and maternal outcome in systemic lupus erythematosus: a prospective study of 103 pregnancies. Rheumatol (Oxford) (2002) 41(6):643–50. 10.1093/rheumatology/41.6.643 12048290

[167] Kaczynski P Bauersachs S Baryla M Goryszewska E Muszak J Grzegorzewski WJ Estradiol-17β-Induced Changes in the Porcine Endometrial Transcriptome In Vivo. Int J Mol Sci (2020) 21(3). 10.3390/ijms21030890 PMC7037416 32019139

[168] Romero R Erez O Maymon E Chaemsaithong P Xu Z Pacora P The maternal plasma proteome changes as a function of gestational age in normal pregnancy: a longitudinal study. Am J Obstet Gynecol (2017) 217(1):67.e1–67.e21. 10.1016/j.ajog.2017.02.037 28263753 PMC5813489

[169] Santoro N Randolph JF Jr Reproductive hormones and the menopause transition. Obstetr Gynecol Clinics North Am (2011) 38(3):455–66. 10.1016/j.ogc.2011.05.004 PMC3197715 21961713

[170] Kim GW Park K Jeong GW Effects of Sex Hormones and Age on Brain Volume in Post-Menopausal Women. J Sex Med (2018) 15(5):662–70. 10.1016/j.jsxm.2018.03.006 29628218

[171] Gambacciani M Levancini M Hormone replacement therapy and the prevention of postmenopausal osteoporosis. Przeglad menopauzalny = Menopause Rev (2014) 13(4):213–20. 10.5114/pm.2014.44996 PMC4520366 26327857

[172] Ronkainen PH Pöllänen E Alén M Pitkänen R Puolakka J Kujala UM Global gene expression profiles in skeletal muscle of monozygotic female twins discordant for hormone replacement therapy. Aging Cell (2010) 9(6):1098–110. 10.1111/j.1474-9726.2010.00636.x 20883525

[173] Cheishvili D Parashar S Mahmood N Arakelian A Kremer R Goltzman D Identification of an Epigenetic Signature of Osteoporosis in Blood DNA of Postmenopausal Women. J Bone Miner Res (2018) 33(11):1980–9. 10.1002/jbmr.3527 29924424

[174] Reppe S Lien TG Hsu YH Gautvik VT Olstad OK Yu R Distinct DNA methylation profiles in bone and blood of osteoporotic and healthy postmenopausal women. Epigenetics (2017) 12(8):674–87. 10.1080/15592294.2017.1345832 PMC5687328 28650214

[175] Maggio M Basaria S Ceda GP Ble A Ling SM Bandinelli S The relationship between testosterone and molecular markers of inflammation in older men. J Endocrinol Invest (2005) 28(11 Suppl Proceedings):116–9. 16760639

[176] Franceschi C Garagnani P Parini P Giuliani C Santoro A Inflammaging: a new immune-metabolic viewpoint for age-related diseases. Nat Rev Endocrinol (2018) 14(10):576–90. 10.1038/s41574-018-0059-4 30046148

[177] Furman D Hejblum BP Simon N Jojic V Dekker CL Thiebaut R Systems analysis of sex differences reveals an immunosuppressive role for testosterone in the response to influenza vaccination. Proc Natl Acad Sci U S A (2014) 111(2):869–74. 10.1073/pnas.1321060111 PMC3896147 24367114

[178] Cundill P Hormone therapy for trans and gender diverse patients in the general practice setting. Aust J Gen Pract (2020) 49(7):385–90. 10.31128/AJGP-01-20-5197 32599993

[179] Klaver M de Blok CJM Wiepjes CM Nota NM Dekker M de Mutsert R Changes in regional body fat, lean body mass and body shape in trans persons using cross-sex hormonal therapy: results from a multicenter prospective study. Eur J Endocrinol (2018) 178(2):163–71. 10.1530/EJE-17-0496 29183889

[180] Arnold AP Chen X What does the “four core genotypes” mouse model tell us about sex differences in the brain and other tissues? Front Neuroendocrinol (2009) 30(1):1–9. 10.1016/j.yfrne.2008.11.001 19028515 PMC3282561

[181] Burgoyne PS Arnold AP A primer on the use of mouse models for identifying direct sex chromosome effects that cause sex differences in non-gonadal tissues. Biol Sex Differ (2016) 7:68. 10.1186/s13293-016-0115-5 27999654 PMC5154145

[182] Chen X McClusky R Chen J Beaven SW Tontonoz P Arnold AP The number of x chromosomes causes sex differences in adiposity in mice. PLoS Genet (2012) 8(5):e1002709. 10.1371/journal.pgen.1002709 22589744 PMC3349739

[183] Chen X Wang L Loh DH Colwell CS Tache Y Reue K Sex differences in diurnal rhythms of food intake in mice caused by gonadal hormones and complement of sex chromosomes. Horm Behav (2015) 75:55–63. 10.1016/j.yhbeh.2015.07.020 26226656 PMC4648666

[184] Ocon A Peredo-Wende R Kremer JM Bhatt BD Significant symptomatic improvement of subacute cutaneous lupus after testosterone therapy in a female-to-male transgender subject. Lupus (2018) 27(2):347–8. 10.1177/0961203317734921 28992799

[185] Zandman-Goddard G Solomon M Barzilai A Shoenfeld Y Lupus erythematosus tumidus induced by sex reassignment surgery. J Rheumatol (2007) 34(9):1938. 17696264

[186] Chan KL Mok CC Development of systemic lupus erythematosus in a male-to-female transsexual: the role of sex hormones revisited. Lupus (2013) 22(13):1399–402. 10.1177/0961203313500550 23897544

[187] Pontes LT Camilo DT De Bortoli MR Santos RSS Luchi WM New-onset lupus nephritis after male-to-female sex reassignment surgery. Lupus (2018) 27(13):2166–9. 10.1177/0961203318800571 30231802

[188] Aranda G Fernández-Rebollo E Pradas-Juni M Hanzu FA Kalko SG Halperin I Effects of sex steroids on the pattern of methylation and expression of the promoter region of estrogen and androgen receptors in people with gender dysphoria under cross-sex hormone treatment. J Steroid Biochem Mol Biol (2017) 172:20–8. 10.1016/j.jsbmb.2017.05.010 28539237

[189] Ackerley CG Billingsley JM Tharp GK Amancha PK Tangpricha V Smith SA Transgender Women on Feminizing Hormone Therapy Demonstrate a Distinct Rectal Mucosal Transcriptome from Cisgender Men. AIDS Res Hum Retroviruses (2020) 36(9):771–4. 10.1089/aid.2020.0061 PMC7482106 32611248

[190] Koeken V de Bree LCJ Mourits VP Moorlag SJ Walk J Cirovic B BCG vaccination in humans inhibits systemic inflammation in a sex-dependent manner. J Clin Invest (2020) 130(10):5591–602. 10.1172/JCI133935 PMC7524503 32692728

[191] Klein SL Poland GA Personalized vaccinology: one size and dose might not fit both sexes. Vaccine (2013) 31(23):2599. 10.1016/j.vaccine.2013.02.070 23579257

